# Depletion of Intracellular Glutamine Pools Triggers *Toxoplasma gondii* Stage Conversion in Human Glutamatergic Neurons

**DOI:** 10.3389/fcimb.2021.788303

**Published:** 2022-01-13

**Authors:** Hironori Bando, Yasuhiro Fukuda, Nina Watanabe, Jeje Temitope Olawale, Kentaro Kato

**Affiliations:** ^1^ Laboratory of Sustainable Animal Environment, Graduate School of Agricultural Science, Tohoku University, Osaki, Japan; ^2^ Department of Biochemistry, Faculty of Science, Federal University Oye-Ekiti, Oye-Ekiti, Ekiti State, Nigeria; ^3^ Department of Biochemistry, School of Science, Federal University of Technology, Akure, Nigeria

**Keywords:** bradyzoite, IFN-γ, iPSC-derived glutamatergic neurons, *Toxoplasma gondii*, human

## Abstract

*Toxoplasma gondii* chronically infects the brain as latent cysts containing bradyzoites and causes various effects in the host. Recently, the molecular mechanisms of cyst formation in the mouse brain have been elucidated, but those in the human brain remain largely unknown. Here, we show that abnormal glutamine metabolism caused by both interferon-γ (IFN-γ) stimulation and *T. gondii* infection induce cyst formation in human neuroblastoma cells regardless of the anti-*T. gondii* host factor nitric oxide (NO) level or Indoleamine 2,3-dioxygenase-1 (IDO1) expression. IFN-γ stimulation promoted intracellular glutamine degradation in human neuronal cells. Additionally, *T. gondii* infection inhibited the mRNA expression of the host glutamine transporters SLC38A1 and SLC38A2. These dual effects led to glutamine starvation and triggered *T. gondii* stage conversion in human neuronal cells. Furthermore, these mechanisms are conserved in human iPSC-derived glutamatergic neurons. Taken together, our data suggest that glutamine starvation in host cells is an important trigger of *T. gondii* stage conversion in human neurons.

## Introduction

*Toxoplasma gondii* is an obligate intracellular protozoan parasite that can infect the nucleated cells of all warm-blooded animals (Boothroyd, 2009; Dubey, 2010); it is estimated that one-third of the world’s population has already been infected (El-On and Peiser, 2003). Once *T. gondii* parasites enter the host, they spread the infection throughout the entire body through the bloodstream by hijacking host immune cells such as macrophages (intracellular parasites) (Bierly et al., 2008; Coombes et al., 2013) or free tachyzoites (extracellular parasites) (Unno et al., 2008). *T. gondii* in many types of tissues grow as tachyzoites, which is the rapidly multiplying form in the acute phase of infection (Dubey, 2009); however, parasite infection in specific organs such as the brain or muscle tissues leads to parasite stage conversion into bradyzoites, which is the slowly multiplying form in the chronic phase of infection that remains throughout the host’s life (Robert-Gangneux and Darde, 2012; Watts et al., 2015). Although anti-*T. gondii* drugs such as pyrimethamine, sulfadiazine, and atovaquone effectively inhibit the number of tachyzoites, they carry the potential risk of inducing parasite stage conversion to the chronic phase of infection (Ferguson et al., 1994; Gormley et al., 1998; Alday and Doggett, 2017). Additionally, there are no curative drugs that can eliminate bradyzoites. Infected immunocompromised people and fetuses are particularly susceptible to serious symptoms, such as toxoplasmosis, hydrocephalus, and chorioretinitis (Frenkel and Remington, 1980; Montoya and Remington, 2008). *T. gondii*-infected healthy people are generally asymptomatic; however, recently it has been revealed that chronic infection with *T. gondii* is a potential cause of various diseases such as depression (Sutterland et al., 2015; Cheng et al., 2020). Therefore, it is important to gain an understanding of the mechanism of *T. gondii* stage conversion to chronic infection.

*T. gondii* bradyzoite differentiation or cyst formation induction models using artificial stress stimulation such as high or low pH and heat shock have been established (Weiss et al., 1995; Lyons et al., 2002), and many kinds of parasite genes related to stage conversion and the molecular mechanisms involved have been clarified (Radke et al., 2013; Waldman et al., 2020). For example, *T. gondii* surface antigen 1 (SAG1), a major surface protein of tachyzoites, is involved in cell adhesion and invasion. SAG1 is a tachyzoite-specific marker because its expression is not detected in bradyzoites (Kasper, 1989). *T. gondii* bradyzoite antigen 1 (BAG1) is a bradyzoite-specific marker (Tomavo et al., 1991), and cyst wall markers [e.g., bradyzoite-specific cyst-wall protein 1 (CST1)], which are markers of bradyzoite differentiation, have also been identified (Zhang and Smith, 1995). Thus, various stage-specific markers have been revealed, and used to confirm parasite differentiation (Mayoral et al., 2020).

Because *T. gondii* stage conversion is a cell-type-specific event, it has been suggested that some host factors play an important role in its induction (Lüder and Rahman, 2017). Mice have been used to reveal the physiological stimulations that play an important role in the induction of bradyzoite differentiation or cyst formation; these studies identified some key host factors that induce parasite stage conversion. For example, treatment with mitochondrial inhibitors such as oligomycin and antimycin A induced bradyzoite differentiation in murine bone marrow-derived macrophages (BMDMs) (Bohne et al., 1994). Cell division autoantigen 1 (CDA-1), which is involved in cell cycle progression, has been shown to trigger bradyzoite differentiation and cyst formation in murine skeletal muscle tissue and human fibroblasts (Radke et al., 2006; Swierzy and Lüder, 2015). Inflammatory cytokines, such as interferon-γ (IFN-γ) and tumor necrosis factor-α (TNF-α), have been shown to be important for the induction of parasite stage conversion in mice (Yap et al., 1998; Tobin et al., 2010). For instance, IFN-γ-dependent nitric oxide (NO) production led to stress, including arginine starvation, which induced parasite stage conversion (Bohne et al., 1994; Fox et al., 2004). However, these mechanisms are not common to all cell types or host types; for example, IFN-γ-dependent induction of bradyzoite formation in human monocytes and human foreskin fibroblasts (HFFs) has not been observed (Bohne et al., 1993; Soête et al., 1994). Thus, the key host factors involved in inducing bradyzoite formation in human cells remain largely unknown.

In the present study, we found that IFN-γ stimulation induced bradyzoite formation in human neuronal cells. We further found that the effects of both IFN-γ-dependent intracellular glutamine degradation by glutaminase and *T. gondii* infection-dependent inhibition of glutamine transporter activation led to glutamine starvation in IFN-γ-stimulated, *T. gondii*-infected human neuroblastoma cells and human iPSC-derived glutamatergic neurons. Taken together, our data demonstrate that glutamine starvation is a key host factor for inducing *T. gondii* stage conversion in human neuronal cells.

## Materials and Methods

### Cell Lines and Parasites

*T. gondii* strains ME49 and Prugniaud were maintained in Vero cells in RPMI (Nacalai Tesque) supplemented with 2% heat-inactivated fetal bovine serum (FBS; JRH Bioscience), 100 U/mL penicillin, and 0.1 mg/mL streptomycin (Nacalai Tesque), as previously described (Ma et al., 2014). HFFs were maintained in RPMI (Nacalai Tesque) supplemented with 2% heat-inactivated FBS (JRH Bioscience), 100 U/mL penicillin, and 0.1 mg/mL streptomycin (Nacalai Tesque). IMR-32 cells were maintained in MEM (Nacalai Tesque) containing 10% heat-inactivated FBS, 1% non-essential amino acids (Nacalai Tesque), 100 U/mL penicillin, and 0.1 mg/mL streptomycin. A172 cells were maintained in DMEM (Nacalai Tesque) containing 10% heat-inactivated FBS, 100 U/mL penicillin, and 0.1 mg/mL streptomycin. U251-MG cells were maintained in EMEM (Nacalai Tesque) containing 10% heat-inactivated FBS, 100 U/mL penicillin, and 0.1 mg/mL streptomycin.

### iPSC-Derived Glutamatergic Neurons

To prepare iPSC-derived glutamatergic neurons, ioGlutamatergic neurons were obtained from Abcam (ab259259). ioGlutamatergic neurons (5.7 × 10^4^) were plated in a 24-well plate containing PDL-Geltrex-coated glass coverslips. On days 0–4, the neurons were cultured in complete glutamatergic neuron medium (CGNM) containing 1 μg/mL doxycycline (Sigma–Aldrich). The CGNM comprised 200 mL of Neurobasal medium (Thermo Fisher), 2 mL of GlutaMAX (100×) (Thermo Fisher), 25 μM 2-mercaptoethanol (Thermo Fisher), 4 mL of B27 (Thermo Fisher), 10 ng/mL NT3 (R&D), and 5 ng/mL BDNF (R&D). On days 5–14 days, the neurons were cultured in CGNM without doxycycline. The medium was changed every 48 h. After 12 days in culture, differentiation of iPSC-derived glutamatergic neurons was confirmed by morphology and gene expression of neuron or glutamatergic neuron-specific markers by use of microscopy and an immunofluorescence assay, respectively.

### Reagents

Antibodies against TUBB3 (66375-1-lg), KGA (20170-1-AP), and VGLUT1 (55491-1-AP) were obtained from Proteintech. Salmon E monoclonal antibody for CST1 staining has been described previously (Tomita et al., 2013). The anti-GAP45 antibody was kindly gifted by Dr. Dominique Soldati-Favre (University of Geneva, Switzerland). Recombinant human IFN-γ (300-02) was obtained from Peprotech. Aminoguanidine hydrochloride (396494) was obtained from Sigma–Aldrich. 1-Methyl-DL-tryptophan (sc-224746) and KGA siRNA (sc-105592) were obtained from Santa Cruz Biotechnology, Inc. CB-839 (Cay-22038) was obtained from Cayman Chemical.

### Bradyzoite Differentiation

Bradyzoite differentiation was confirmed by the gene expression pattern of *SAG1* and *BAG1*, and by staining of the cyst wall protein CST1. HFFs, IMR-32, A172, U-251 MG cells, or iPSC-derived glutamatergic neurons were cultured and infected with *T. gondii* [multiplicity of infection (MOI) = 0.5]. For alkaline induction, the culture medium was changed to non-induction medium (pH 7.2) or induction medium (pH 8.2) at 2 h post-infection. For IFN-γ induction, the cells were stimulated with or without IFN-γ (10 ng/ml) at 2 h post-infection. Infected host cells were incubated for 24–72 h under low CO_2_ conditions. The cyst wall was stained by using an anti-CST1 antibody, and CST1-positive vacuoles were defined as bradyzoite differentiated parasitophorous vacuoles. Quantitative measurements of the cyst wall-positive vacuole rates were performed by counting at least 100 vacuoles per sample.

### Quantitative RT-PCR

Total RNA from cells or parasites was extracted by using an RNA basic kit (Nippon genetics), and cDNA was synthesized by using Verso Reverse transcription (Thermo Fisher). Quantitative RT-PCR was performed with a Thermal Cycler Dice Real Time PCR System (Takara) using the Go-Taq Real-Time PCR system (Promega). The values were normalized to glyceraldehyde 3-phosphate dehydrogenase (*GAPDH*) for human cells or *Tubulin* or *Actin* for *T. gondii* in each sample. The primer sequences are listed in **S1 Table**.

### Ion Chromatography (IC)

IMR-32 cells or A172 cells were plated in a 6-well plate, and then infected or non-infected cells were incubated for 48 h. The culture supernatants were then collected and filtrated through a 0.4-μm filter membrane before being analyzed. The concentrations of 11 major ions $\left(F^{-},\;Cl^{-},\;Br^{-},\;NO_{2}^{-},\;PO_{4}^{3-},\;SO_{4}^{2-},NA^{+},\;NH_{4}^{+},\;K^{+},\;Mg^{2+},\;Ca^{2+}\right)$ were measured by using the HIC-20A SUPER ion chromatography system (Shimazu).

### Measurement of Kynurenine

The kynurenine concentration in the culture medium was measured by using the Ehrlich reagent method (Braun et al., 2005). Briefly, 70 μL of culture supernatant was mixed with 35 μL of 30% trichloroacetic acid, and centrifuged at 8,000 × *g* for 5 min. Then, 75 μL of the supernatant was added to an equal volume of Ehrlich reagent (0.8% p-dimethylaminobenzaldehyde in acetic acid) in a 96-well plate, and the absorbance was read at 490 nm. The values were determined by using a standard curve with defined concentrations of kynurenine (Sigma–Aldrich).

### Measurement of Glutamine

IMR-32 cells or iPSC-derived glutamatergic neurons were plated in a 96-well plate, and then infected and non-infected cells were incubated for 24 or 48 h. The concentration of extracellular and intracellular glutamine was measured by using the Glutamine/Glutamate-Glo™ Assay (Promega) and a GloMax Navigator Microplate Luminometer (Promega) according to the manufacturer’s instructions.

### Immunofluorescence Assays

HFFs, IMR-32, A172, and U-251 MG cells, or iPSC-derived glutamatergic neurons were cultured on glass coverslips and infected with *T. gondii* (MOI = 0.5) for the indicated time. The cells were then fixed in PBS containing 4% paraformaldehyde for 15 min at room temperature. Cells were permeabilized with PBS containing 0.1% Triton X-100 for 5 min, and then blocked with 2% FBS in PBS for 1 h at room temperature. Next, the cells were incubated with the indicated primary antibodies for 1 h at 37°C, followed by incubation with Alexa 488- or Alexa 594-conjugated secondary antibodies (Molecular Probes) and DAPI for 1 h at 37°C in the dark. Finally, coverslips were mounted onto glass slides with ProLong Glass Antifade Mountant (Invitrogen) and analyzed by using a BZ-X810 All-in-one Fluorescence Microscope (Keyence).

### Inhibitor Treatment

IMR-32 cells were pre-treated with Nω-Propyl-L-Arginine hydrochloride (2 μM) or 1-methyl-DL-tryptophan (1 mM) for 3 h, and then infected with the parasite. The culture medium was changed 2 h post-infection, and fresh medium containing 50 ng/mL IFN-γ and Nω-Propyl-L-Arginine hydrochloride (2 μM) or 1-Methyl-DL-tryptophan (1 mM) was added for 24–72 h.

### Plaque Assay

HFF cells were pre-treated with CB-839 (2 μM) for 3 h, and then infected with the parasite. The culture medium was changed 2 h post-infection, and fresh medium containing 50 ng/mL IFN-γ with or without CB-839 (2 μM) was added for 5 days. Cells were fixed with 4% paraformaldehyde for 30 min at room temperature, then washed with PBS and stained with 0.1% crystal violet (CV) for 10 min. Images were analyzed by using Image J.

### Western Blot Analyses

Cells were lysed in a lysis buffer (0.5% Nonidet P-40, 150 mM NaCl, and 20 mM Tris-HCl, pH 7.5) containing a protease inhibitor cocktail (Roche). The cell lysates were separated by SDS-PAGE and transferred to polyvinylidene difluoride membranes (Immobilon-P, Millipore) and subjected to Western blot analyses as described previously (Bando et al., 2019).

### siRNA Transfection and Parasite Infection

ioGlutamatergic neurons (5.7 × 10^4^) were plated in a 24-well plate and developed to iPSC-derived glutamatergic neurons. Ten days after development, KGA siRNA was transfected by using Lipofectamine 2000 (Invitrogen, Carlsbad, CA) according to the manufacturer’s instructions. At 24 h post-lipofection, the wells were washed and then incubated for an additional 24 h. KGA siRNA-transfected, iPSC-derived glutamatergic neurons were infected with *T. gondii* (MOI = 0.5) for 2 h, and then stimulated with or without IFN-γ (10 ng/ml). Infected host cells were incubated for 24–72 h under low CO_2_ conditions.

### Statistical Analyses

All statistical analyses were performed by using Excel (Microsoft) or Prism 8 (GraphPad). All experimental points and *n* values represent an average of three biological replicates (three independent experiments). The statistical significance of differences in mean values was analyzed by using an unpaired two-tailed Student’s t-test. *p* < 0.05 was considered statistically significant.

## Results

### IFN-γ Stimulation Induces Stage Conversion in Type II Strains of *T. gondii* in Human Neuroblastoma Cells

**Figure 1:**
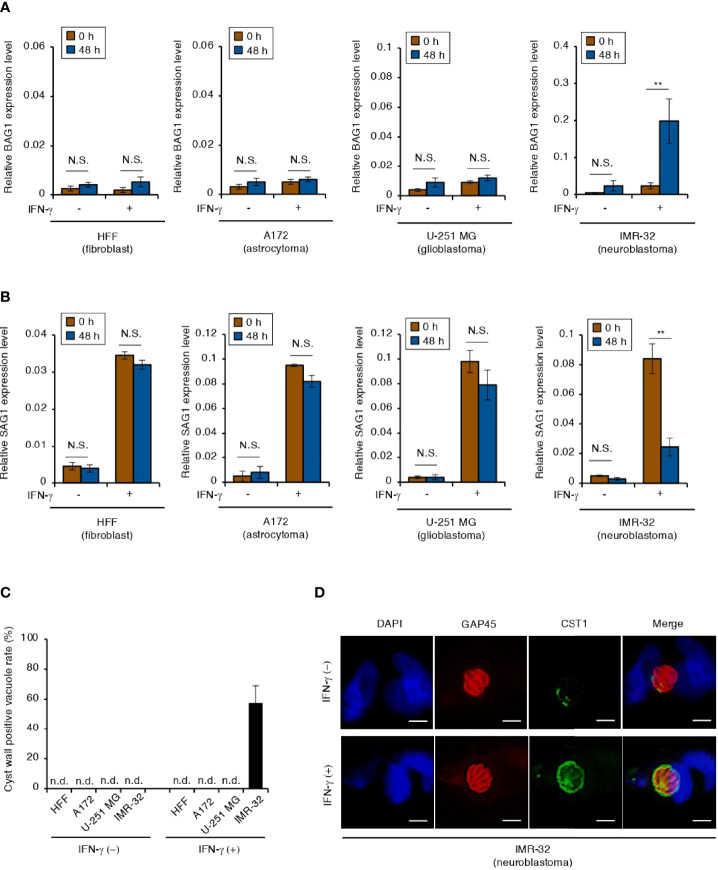
IFN-γ stimulation induces *T. gondii* stage conversion in human neuroblastoma cells. **(A, B)** HFFs, A172, U-251 MG, and IMR-32 cells infected with *T. gondii* ME49 were untreated or treated with IFN-γ and incubated for 0 or 48 h. Then, the *BAG1* **(A)** or *SAG1* **(B)** mRNA level was analyzed by use of quantitative RT-PCR. **(C, D)** IMR-32 cells infected with *T. gondii* strain ME49 were untreated or treated with IFN-γ. Cyst wall formation was assessed by IFA at 72 hours post-infection. **(C)** The percentage of CST1-positive vacuoles was determined. **(D)** Representative IFA images of *T. gondii* GAP45 (red) and CST1 (green); nuclei were stained with DAPI (blue). Scale bars correspond to 5 μm. Data are representative of three independent experiments. Indicated values are means ± SD (three biological replicates per group from three independent experiments) **(A–C)**. **p < 0.01; N.S., not significant; n.d., not detected; Student’s *t*-test.

The physiological conditions that induce *T. gondii* stage conversion in human brain cells are unclear. Therefore, we tested the effect of IFN-γ on the expression of the bradyzoite-specific gene *BAG1* or the tachyzoite-specific gene *SAG1* by using the *T. gondii* Type II ME49 strain in the following human brain cell lines: astrocytoma cell line (A172), glioblastoma cell line (U-251 MG), and neuroblastoma cell line (IMR-32) (**Figures 1A, B**). Although alkaline stress (pH 8.2) induced *BAG1* gene expression in all cell lines tested (**Figure S1A**), IFN-γ-dependent upregulation of *BAG1* gene expression was observed in IMR-32 cells, but not in A172 or U-251 MG cells (**Figure 1A**). In addition, we found that *SAG1* gene expression was downregulated in IMR-32 cells (**Figure 1B**). *T. gondii* cyst wall CST1 formation around bradyzoites in IMR-32 cells was found only in IFN-γ-stimulated cells (**Figures 1C, D**). These phenomena were also observed with the Type II *T. gondii* Prugniaud strain (**Figures S1B, C**). These results indicate that Type II *T. gondii* differentiate into bradyzoites and form a cyst wall in response to IFN-γ stimulation in human neuroblastoma cells.

### IFN-γ-Dependent Bradyzoite Differentiation in Human Neuroblastoma Cells Does Not Rely on NO Production

**Figure 2:**
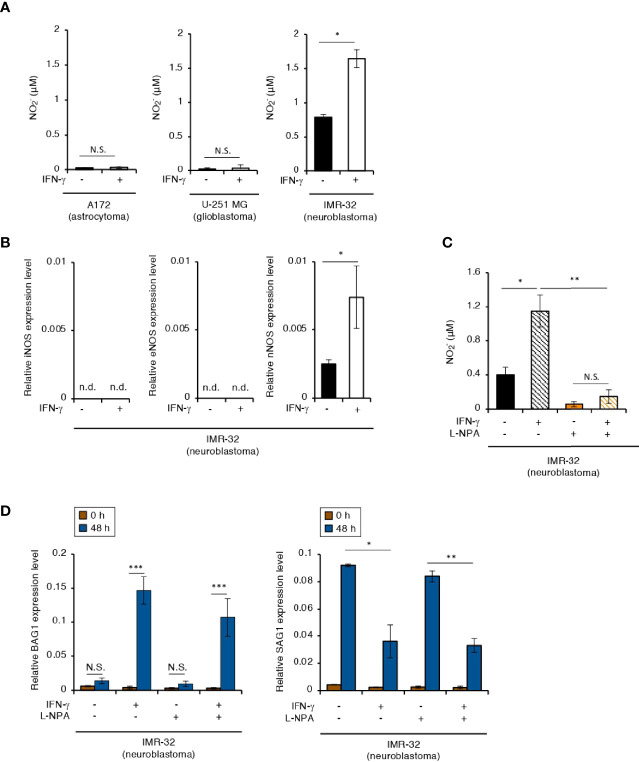
NO concentration plays no role in the IFN-γ-dependent *T. gondii* stage conversion in human neuroblastoma cells. **(A)** A172, U-251 MG, and IMR-32 cells were untreated or treated with IFN-γ and incubated for 0 or 48 h. The level of NO_2_ released into the culture supernatant was measured by IC. **(B)** IMR-32 cells were untreated or treated with IFN-γ and incubated for 24 h. The *iNOS*, *eNOS*, and *nNOS* mRNA levels were analyzed by use of quantitative RT-PCR. **(C)** IMR-32 cells were untreated or treated with IFN-γ and/or aminoguanidine and incubated for 48 h. The level of NO_2_ released into the culture supernatant was measured by IC. **(D)** IMR-32 cells infected with *T. gondii* ME49 were untreated or treated with IFN-γ and/or aminoguanidine and incubated for 48 h. Then, the *BAG1* or *SAG1* mRNA level were analyzed by use of quantitative RT-PCR. Values are means ± SD (three biological replicates per group from three independent experiments) **(A–D)**. *p < 0.05, **p < 0.01, ***p < 0.001; N.S., not significant; Student’s *t*-test. n.d., not detected.

IFN-γ-induced bradyzoite differentiation in murine BMDMs depends on NO production (Bohne et al., 1994). In our study, an IFN-γ-dependent increase in NO production was detected in IMR-32 cells, but not in A172 or U251-MG cells (**Figure 2A**). Humans have three isoforms of nitric oxide synthase (NOS): inducible NOS (iNOS), epithelial NOS (eNOS), and neuronal NOS (nNOS) (Umar and van der Laarse, 2010). We therefore tested which NOS isoform is important for NO production in IFN-γ-stimulated IMR-32 cells (**Figure 2B**). We detected *nNOS* gene expression in IMR-32 cells but not in A172 cells in an IFN-γ-dependent manner (**Figure 2B** and **Figure S2A**). Then, we tested the effect of IFN-γ-dependent NO production on bradyzoite differentiation in IMR-32 cells by using Nω-propyl-L-arginine (L-NPA), a highly selective nNOS inhibitor (**Figures 2C, D**). Although L-NPA inhibited NO production in IFN-γ-stimulated IMR-32 cells (**Figure 2C**), there was no difference in *BAG1* or *SAG1* gene expression in the presence or absence of L-NPA (**Figure 2D**). These results suggest that NO production does not play a key role in the induction of bradyzoite differentiation in IFN-γ-stimulated human neuroblastoma cells.

### Indoleamine 2,3-Dioxygenase-1 (IDO1)-Dependent Tryptophan Starvation Does Not Influence IFN-γ-Induced Bradyzoite Differentiation in Human Neuroblastoma Cells

**Figure 3:**
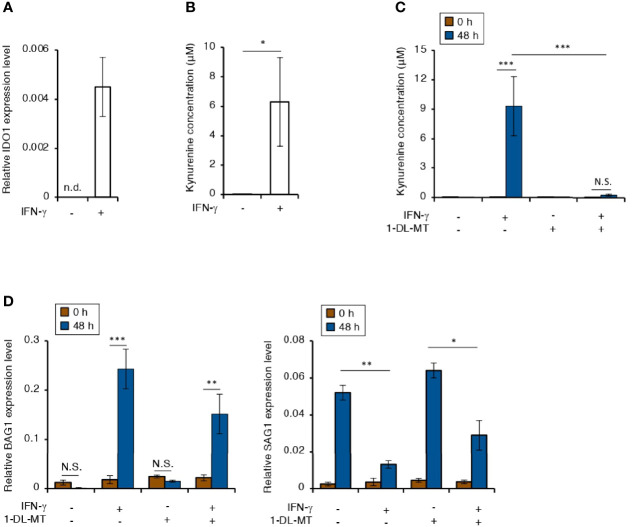
Tryptophan degradation plays no role in the IFN-γ-dependent *T. gondii* stage conversion in human neuroblastoma cells. **(A)** IMR-32 cells were untreated or treated with IFN-γ and incubated for 24 h. The *IDO1* mRNA level was analyzed by use of quantitative RT-PCR. **(B)** IMR-32 cells were untreated or treated with IFN-γ and incubated for 48 h. The level of kynurenine released into the culture supernatant was measured by ELISA. **(C)** IMR-32 cells were untreated or treated with IFN-γ and/or 1-DL-MT and incubated for 48 h. The level of kynurenine released into the culture supernatant was measured by ELISA. **(D)** IMR-32 cells infected with *T. gondii* ME49 were untreated or treated with IFN-γ and/or 1-DL-MT and incubated for 0 or 48 h. Then, the *BAG1* or *SAG1* mRNA level was analyzed by use of quantitative RT-PCR. Values are means ± SD (three biological replicates per group from three independent experiments) **(A–D)**. *p < 0.05, **p < 0.01, ***p < 0.001; N.S., not significant; Student’s *t*-test.

IFN-γ stimulation induces IDO1-dependent tryptophan starvation in various human cells (Bando et al., 2018a). Therefore, we tested the effect of IFN-γ stimulation on IDO1 expression in IMR-32 cells (**Figures 3A, B**). *IDO1* gene expression was induced in IFN-γ-stimulated IMR-32 cells (**Figure 3A**). Furthermore, kynurenine, which is a metabolite in the IDO pathway, was detected in the culture medium of IFN-γ-stimulated IMR-32 cells (**Figure 3B**). Then, we examined the effect of *IDO1* gene expression on bradyzoite differentiation in IMR-32 cells treated with 1-methyl-DL-tryptophan (1-DL-MT), which is an IDO1 inhibitor (**Figures 3C, D**). Although 1-DL-MT treatment inhibited IDO1 activity (**Figure 3C**), there was no difference in the *BAG1* or *SAG1* gene expression pattern (**Figure 3D**). These results suggest that IDO1-dependent tryptophan starvation stress does not affect bradyzoite differentiation in IFN-γ-stimulated human neuroblastoma cells.

### The Concentration of the Metabolic Product NH_4_ in the Culture Medium Is Increased by IFN-γ Stimulation in Human Neuroblastoma Cells

IFN-γ stimulation induced bradyzoite differentiation only in neuroblastoma cells (**Figure 1A**). Therefore, to elucidate the neuronal cell-specific response to IFN-γ stimulation, we compared the IFN-γ-inducible gene expression pattern of predicted-anti-*T. gondii* response genes in A172 and IMR-32 cells (**Figures S2A, B**). We did not find an IMR-32 cell-specific gene expression pattern other than that of *nNOS* (**Figures S2A, B**). Next, we compared the metabolic profiles by using ion chromatography (**Figures S3A, B**). We found that $NO_{2}^{-}$ and $NH_{4}^{+}$ were specifically high in the culture medium of IFN-γ-stimulated IMR-32 cells (**Figures S3A, B**). This IFN-γ-dependent *nNOS* gene expression and NO production confirmed our previous results (**Figures 2A, B**). Therefore, we focused on $NH_{4}^{+}$ as a host factor candidate involved in the induction of bradyzoite differentiation and cyst formation.

### The Intracellular Glutamine Concentration Is Limited in IFN-γ-Stimulated Human Neuroblastoma Cells by *T. gondii* Infection

**Figure 4:**
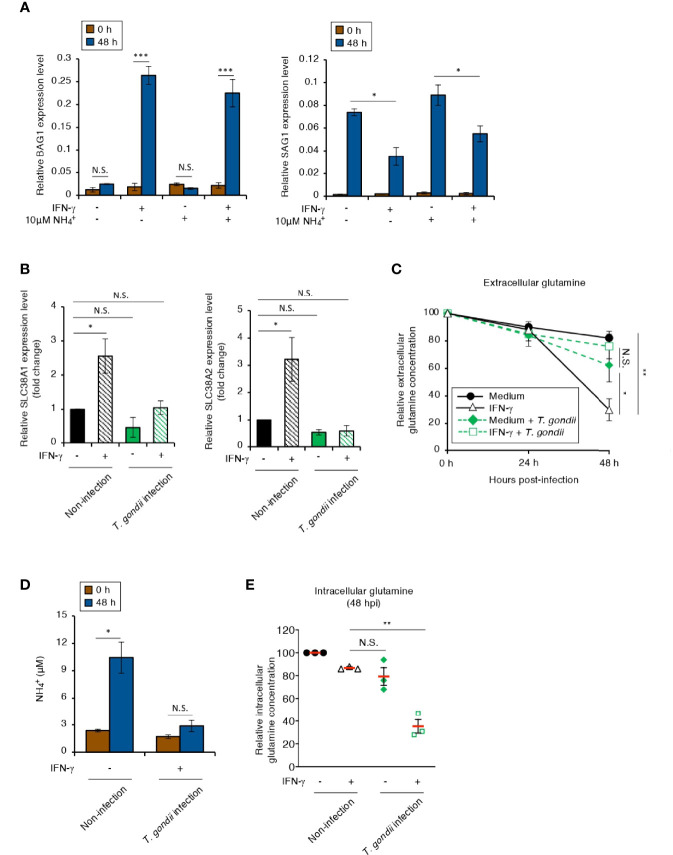
The intracellular glutamine concentration decreases in an IFN-γ-stimulated, *T. gondii*-infected human neuroblastoma cells. **(A)** IMR-32 cells infected with *T. gondii* ME49 were untreated or treated with IFN-γ and/or $NH_{4}^{+}$ and incubated for 0 or 48 h. The *BAG1* or *SAG1* mRNA level was analyzed by use of quantitative RT-PCR. **(B)** IMR-32 cells uninfected or infected with *T. gondii* ME49 were untreated or treated with IFN-γ and incubated for 0 or 48 h. The *SLC38A1* and *SLC38A2* mRNA levels were analyzed by use of quantitative RT-PCR. **(C)** IMR-32 cells uninfected or infected with *T. gondii* ME49 were untreated or treated with IFN-γ. The glutamine level in the culture supernatant at 0, 24, and 48 h after parasite infection was measured by ELISA. **(D)** IMR-32 cells uninfected or infected with *T. gondii* ME49 were untreated or treated with IFN-γ and incubated for 0 or 48 h. The amount of $NH_{4}^{+}$ released into the culture supernatant was measured by IC. **(E)** IMR-32 cells uninfected or infected with *T. gondii* ME49 were untreated or treated with IFN-γ. The level of intracellular glutamine at 48 h after parasite infection was measured by ELISA. Values are means ± SD (three biological replicates per group from three independent experiments) **(A–E)**. *p < 0.05, **p < 0.01, ***p < 0.001; N.S., not significant; Student’s t-test.

NH_4_ accumulation is toxic to many cell types and activates several stress responses (Braissant et al., 2013; Wang et al., 2018). Hence, we examined the effect of a high concentration of NH_4_ in the culture medium on *BAG1* or *SAG1* gene expression in IMR-32 cells (**Figure 4A**). There was no difference in either gene expression pattern in IMR-32 cells cultured with or without a high concentration of NH_4_ (**Figure 4A**). It has been reported that most of the NH_4_ produced in the brain is derived from glutamine metabolism, which includes glutamine influx and glutaminase degradation (Bak et al., 2006). Therefore, we next examined the IFN-γ-dependent expression of the main glutamine transporters: solute carrier family 38 members A1 and A2 (*SLC38A1* and *SLC38A2*) (Varoqui et al., 2000; González-González et al., 2005), in IMR-32 cells (**Figure 4B**). Although IFN-γ induced *SLC38A1* and *SLC38A2* gene expression in non-infected cells, this expression was inhibited in *T. gondii*-infected cells (**Figure 4B**), suggesting that *T. gondii* infection suppressed IFN-γ-dependent glutamine influx in IMR-32 cells. We next examined the glutamine dynamics in the culture medium (extracellular) (**Figures 4C, D**) and in the IMR-32 cells (intracellular) (**Figure 4E**). The extracellular glutamine concentration in the culture medium of IFN-γ-stimulated, *T. gondii*-infected IMR-32 cells was higher than that of IFN-γ-stimulated, non-infected IMR-32 cells (**Figure 4C**). Furthermore, the IFN-γ-induced NH_4_ increase in the culture medium was inhibited by *T. gondii* infection (**Figure 4D**). In contrast, the intracellular glutamine concentration in IFN-γ-stimulated, *T. gondii*-infected IMR-32 cells was significantly reduced compared with that in IFN-γ-stimulated, non-infected IMR-32 cells (**Figure 4E**). These results suggest that *T. gondii* infection leads to intracellular glutamine starvation *via* suppression of glutamine transporter activity in human neuroblastoma cells.

### Glutaminase Activity Directly Affects IFN-γ-Dependent *T. gondii* Stage Conversion in Human Neuroblastoma Cells

**Figure 5:**
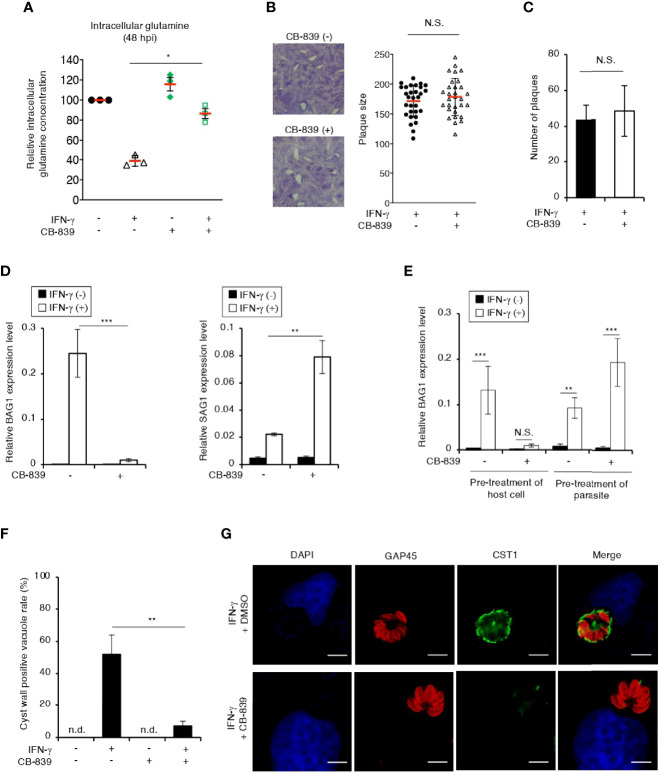
IFN-γ-dependent *T. gondii* stage conversion is suppressed by treatment with a glutaminase inhibitor in human neuroblastoma cells. **(A)** IMR-32 cells infected with *T. gondii* ME49 were untreated or treated with IFN-γ and/or CB-839. The level of intracellular glutamine at 48 h after parasite infection was measured by ELISA. **(B, C)** HFF cells infected with *T. gondii* ME49 were treated with IFN-γ and/or CB-839. Plaques were stained at 4 days post-infection. **(B)** Plaque sizes were measured by using the Image J software. **(C)** Plaque numbers of individual strains were counted. **(D)** IMR-32 cells infected with *T. gondii* ME49 were untreated or treated with IFN-γ and/or CB-839 and incubated for 0 or 48 h. The *BAG1* or *SAG1* mRNA level at 48 h after parasite infection was analyzed by use of quantitative RT-PCR. **(E)** IMR-32 cells or *T. gondii* ME49 were pretreated with CB-839 for 3 h, and then washed before infection. CB-839-pretreated or untreated IMR-32 cells were infected with pre-treated or untreated *T. gondii* ME49. The *BAG1* mRNA levels at 48 h after parasite infection were analyzed by use of quantitative RT-PCR. **(F, G)** IMR-32 cells infected with *T. gondii* ME49 were treated with IFN-γ and/or CB-839. Cyst wall formation was assessed by IFA at 72 h post-infection. **(F)** The percentage of CST1-positive vacuoles was determined. **(G)** Representative IFA images of *T. gondii* GAP45 (red) and CST1 (green); nuclei were stained with DAPI (blue). Scale bars correspond to 5 μm. Data are representative of three independent experiments. Values are means ± SD (three biological replicates per group from three independent experiments) **(A–F)**. *p < 0.01, **p < 0.01, ***p < 0.001; N.S., not significant; n.d., not detected; Student’s *t*-test.

Glutamine serves as a bioenergetic substrate for *T. gondii* growth (Blume et al., 2009), suggesting the possibility that glutamine starvation triggers *T. gondii* stage conversion. We showed that bradyzoite differentiation is not induced in unstimulated IMR-32 cells (**Figure 1**); therefore, we hypothesized that pharmacological inhibition of IFN-γ-dependent glutamine degradation may prevent glutamine starvation and thereby suppress IFN-γ-dependent *T. gondii* stage conversion in IMR-32 cells. To test this hypothesis, we examined the effect of CB-839, a selective glutaminase inhibitor (Xu et al., 2019), on the intracellular glutamine concentration (**Figure 5A**). We found that CB-839 treatment restored the intracellular glutamine concentration in IFN-γ-stimulated, *T. gondii*-infected IMR-32 cells (**Figure 5A**). Next, we examined the effect of CB-839 treatment on the parasite by using a plaque assay, and found that CB-839 treatment had no effect on the parasite plaque size or number (**Figures 5B, C**). Then, we examined the effect of CB-839 treatment on IFN-γ-dependent bradyzoite differentiation (**Figures 5D, E**). We found that IFN-γ-induced *BAG1* gene expression was suppressed in IFN-γ-stimulated IMR-32 cells treated with CB-839 (**Figure 5D**). Importantly, pretreating *T. gondii* with CB-839 did not affect the IFN-γ-induced *BAG1* gene expression (**Figure 5E**), suggesting that the effect of CB-839 treatment was the result of the inhibition of the host glutaminase activity. Furthermore, we examined the effect of CB-839 on *T. gondii* cyst wall CST1 formation (**Figures 5F, G**). We found that CB-839 treatment inhibited IFN-γ-induced cyst wall CST1 formation (**Figures 5F, G**). These results suggest that IFN-γ-induced intracellular glutamine starvation triggers *T. gondii* stage conversion in human neuroblastoma cells.

### *T. gondii* Stage Conversion Is Induced in Human iPSC-Derived Glutamatergic Neurons by IFN-γ-Dependent Intracellular Glutamine Starvation

**Figure 6:**
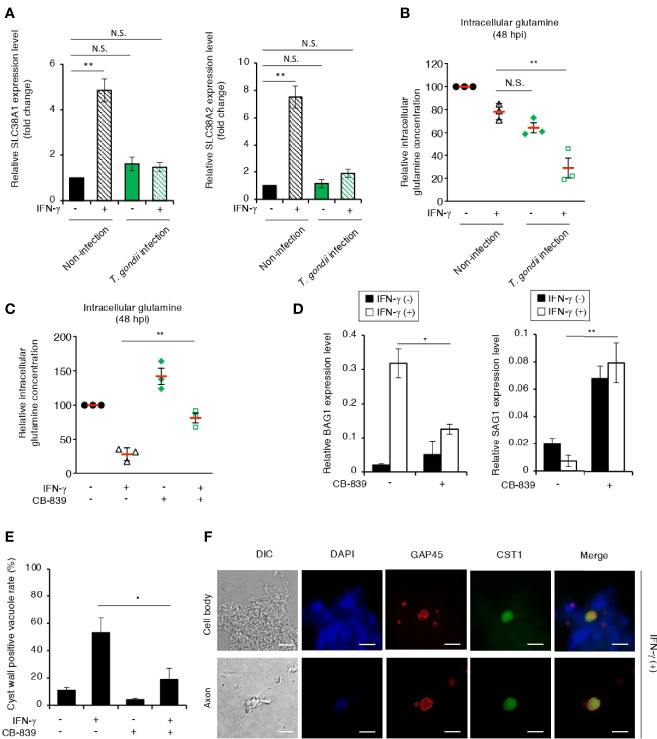
*T. gondii* stage conversion is accelerated by IFN-γ-dependent glutamine starvation in human iPSC-derived glutamatergic neurons. **(A)** Human iPSC-derived glutamatergic neurons uninfected or infected with *T. gondii* ME49 were untreated or treated with IFN-γ and incubated for 24 h. The *SLC38A1* or *SLC38A2* mRNA level was analyzed by use of quantitative RT-PCR. **(B)** Human iPSC-derived glutamatergic neurons uninfected or infected with *T. gondii* ME49 were untreated or treated with IFN-γ. The level of intracellular glutamine at 48 h after parasite infection was measured by ELISA. **(C)** Human iPSC-derived glutamatergic neurons infected with *T. gondii* ME49 were untreated or treated with IFN-γ and/or CB-839. The level of intracellular glutamine at 48 h after parasite infection was measured by ELISA. **(D)** Human iPSC-derived glutamatergic neurons infected with *T. gondii* ME49 were untreated or treated with IFN-γ and/or CB-839. The *BAG1* or *SAG1* mRNA level at 48 h after parasite infection was analyzed by use of quantitative RT-PCR. **(E, F)** Human iPSC-derived glutamatergic neurons infected with *T. gondii* ME49 were treated with IFN-γ. Cyst wall formation in the cell bodies or axons was assessed by IFA at 72 h post-infection. **(E)** The percentage of CST1-positive vacuoles was determined. **(F)** Representative IFA images of *T. gondii* GAP45 (red) and CST1 (green); the nuclei were stained with DAPI (blue). Scale bars correspond to 10 μm. Data are representative of three independent experiments. Values are means ± SD (three biological replicates per group from three independent experiments) **(A–E)**. *p < 0.05, **p < 0.01, N.S., not significant; Student’s *t*-test.

**Figure 7:**
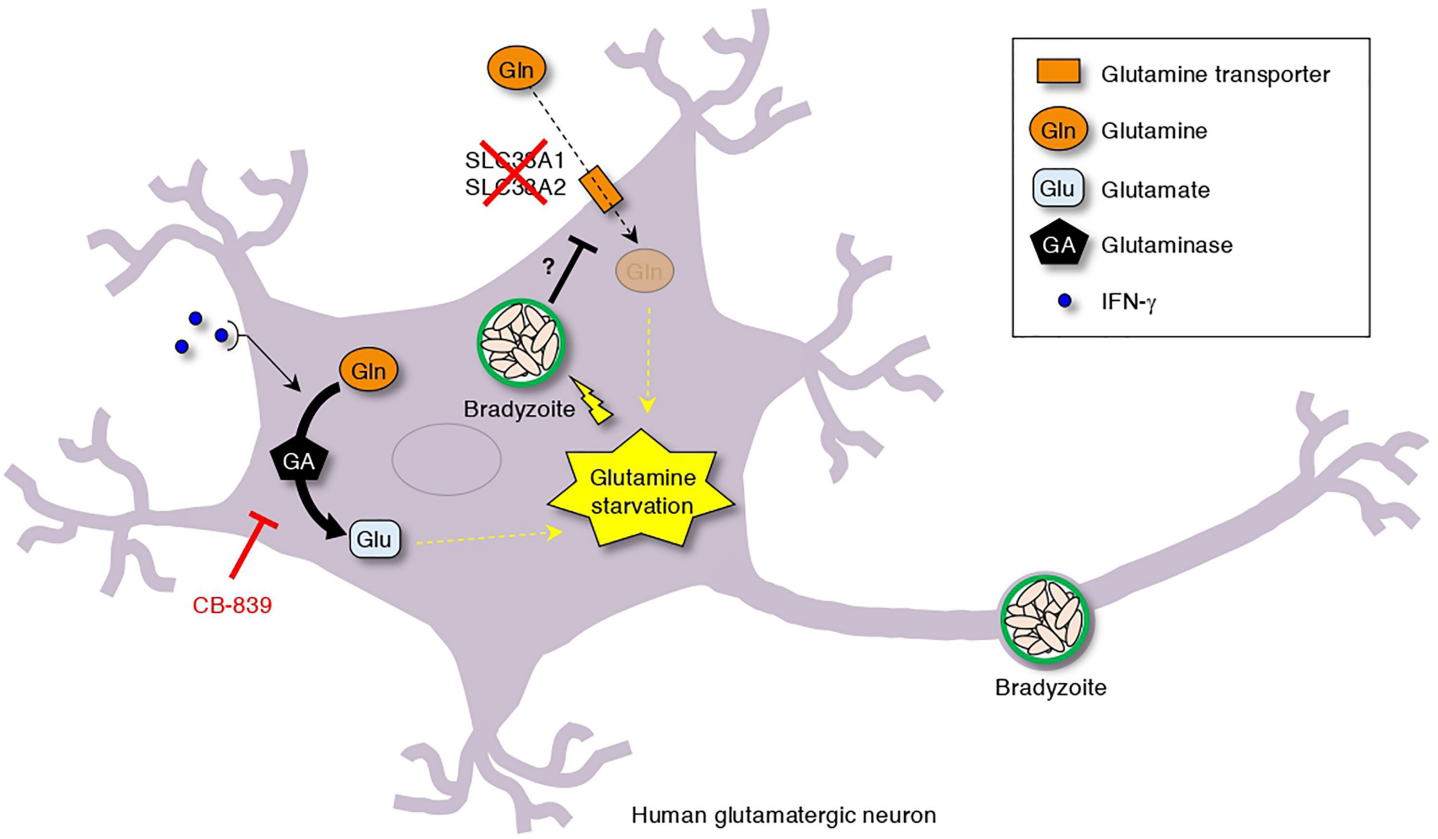
Simplified scheme of *T. gondii* stage conversion in human neuronal cells. The dual effect of *T. gondii* infection-dependent inhibition of glutamine transporter activation and IFN-γ-dependent glutamine degradation cause glutamine starvation, which triggers IFN-γ-dependent *T. gondii* stage conversion in human neuronal cells. As shown, CB-839 treatment can prevent IFN-γ-dependent *T. gondii* stage conversion.

Glutamatergic neurons produce glutamate, which is one of the most common excitatory neurotransmitters in the CNS (Zeng and Sanes, 2017). To confirm the glutamine starvation-induced *T. gondii* stage conversion in glutamatergic neurons, we differentiated glutamatergic neurons from human induced pluripotent stem cells (iPSCs) (**Figures S4A, B**). Axon elongation was evident in a healthy neuronal culture of iPSC-derived neurons 12 days after differentiation induction (**Figure S4A**). In addition, the neuronal marker tubulin beta 3 class III (TUBB3) (Ferreira and Caceres, 1992) and the specific biochemical marker of glutamatergic neurons and glutamatergic synapses vesicular glutamate transporter 1 (VGLUT1) (El Mestikawy et al., 2011) were detected in the iPSC-derived neurons (**Figure S4B**), suggesting that most of the iPSC-derived neurons differentiated into glutamatergic neurons. To confirm the effect of IFN-γ stimulation on *T. gondii* stage conversion, we examined the mRNA expression of the glutamine transporters *SLC38A1* and *SLC38A2* in iPSC-derived glutamatergic neurons (**Figure 6A**). The expression levels of *SLC38A1* and *SLC38A2* were upregulated by IFN-γ stimulation; however, they were inhibited by *T. gondii* infection in iPSC-derived glutamatergic neurons (**Figure 6A**). We next examined the effect of parasite infection on intracellular glutamine concentration in iPSC-derived glutamatergic neurons (**Figure 6B**). The intracellular glutamine concentration in IFN-γ-stimulated, *T. gondii*-infected iPSC-derived glutamatergic neurons was significantly reduced compared with that in IFN-γ-stimulated, non-infected iPSC-derived glutamatergic neurons (**Figure 6B**). Then, we examined the effect of CB-839 on the intracellular glutamine concentration and *BAG1* and *SAG1* gene expression in iPSC-derived glutamatergic neurons (**Figures 6C, D**). We found that CB-839 treatment restored the intracellular glutamine concentration in IFN-γ-stimulated, *T. gondii*-infected iPSC-derived glutamatergic neurons (**Figure 6C** **).** In addition, downregulation of *BAG1* or upregulation of *SAG1* gene expression were observed in response to CB-839 treatment compared to non-treatment in IFN-γ-stimulated, *T. gondii*-infected iPSC-derived glutamatergic neurons (**Figure 6D**). Finally, we examined the effect of CB-839 on *T. gondii* cyst wall CST1 formation in iPSC-derived glutamatergic neurons (**Figures 6E, F**). We found that CB-839 treatment inhibited IFN-γ-induced cyst wall CST1 formation (**Figures 6E, F**). Furthermore, inhibition of IFN-γ-induced *T. gondii* stage conversion was also observed in glutaminase knockdown iPSC-derived glutamatergic neurons (**Figure S5**). These results indicate that disruption of glutamine metabolism by both *T. gondii* infection and IFN-γ stimulation lead to glutamine starvation, triggering *T. gondii* stage conversion in human neuronal cells, including glutamatergic neurons (**Figure 7**).

## Discussion

In the present study, we demonstrated that IFN-γ induces *T. gondii* stage conversion in human neuronal cells and that the mechanism involves glutamine starvation caused by IFN-γ-dependent activation of glutamine metabolism and *T. gondii* infection-induced inhibition of glutamine transporter activation in host cells.

Although IFN-γ is essential for the anti-*T. gondii* host immune response, its molecular mechanism is somewhat different in mice and humans (Ohshima et al., 2014; Bando et al., 2018a). For example, IFN-γ-inducible GTPase-dependent destruction of parasitophorous vacuoles plays an important role in the IFN-γ-dependent anti-*T. gondii* activity in mice (Yamamoto et al., 2012). However, IFN-γ-induced, IDO1-dependent tryptophan degradation has been shown to be important in the anti-*T. gondii* response in humans (Pfefferkorn et al., 1986; Bando et al., 2018a). These findings suggest that stress responses induced by parasites and caused by IFN-γ also differ between mice and humans. Previous reports have shown that the differences in *T. gondii* stress responses are connected to stage conversion induced by IFN-γ stimulation (Bohne et al., 1994; Tobin et al., 2010); however, the role of IFN-γ in *T. gondii* stage conversion in humans is unknown. In this study, we found that IFN-γ-dependent induction of stage conversion of Type II *T. gondii* occurred in neuroblastoma cells and human iPSC-derived glutamatergic neurons. Although previous studies report spontaneous cyst formation of *T. gondii* in human primary neurons and murine skeletal muscle cells (Halonen et al., 1996; Ferreira-Da-Silva Mda et al., 2009), we found that spontaneous stage conversion occurred in iPSC-derived glutamatergic neurons but not in IMR-32 cells, which is consistent with previous studies. However, the stage conversion rate was enhanced by IFN-γ stimulation in iPSC-derived glutamatergic neurons, suggesting that IFN-γ has a role in accelerating stage conversion in iPSC-derived glutamatergic neurons. Investigating the differences between iPSC-derived glutamatergic neurons and IMR-32 cells may be important for determining the cell-type specificity of *T. gondii* stage conversion or the mechanisms of spontaneous stage conversion.

In previous reports, NO-dependent arginine starvation was shown to be important for bradyzoite formation in mouse BMDMs (Fox et al., 2004). In contrast, in the present study, we found that IFN-γ-dependent *T. gondii* stage conversion in neuronal cells was independent of NO production. The reason for this contradiction may be that the IFN-γ-dependent NO production levels are different between mouse cells and human cells. The NO concentration in response to IFN-γ stimulation of mouse cells has been reported to reach 100 μM (Gomez-Marin, 2000), whereas that in human cells is less than 10 μM according to a previous study (Bando et al., 2018b) and this study. Therefore, it is likely that NO is produced in human neuronal cells upon IFN-γ stimulation, but not to a sufficient extent to induce *T. gondii* stage conversion. We also found that IDO1-dependent tryptophan degradation was not associated with the induction of IFN-γ-dependent *T. gondii* stage conversion in human neuronal cells. Although we showed that tryptophan degradation by IDO1 occurs in neuronal cells upon IFN-γ stimulation before *T. gondii* infection (pre-treatment conditions), we have previously shown that *T. gondii* inhibits IDO1 activity *via* the parasite virulence factor TgIST upon IFN-γ stimulation after *T. gondii* infection (post-treatment conditions) (Bando et al., 2018a). *T. gondii* must inhibit IFN-γ-dependent anti-*T. gondii* immune responses to survival, but it also must activate the IFN-γ-dependent host metabolism to change its life-stage. Therefore, the counterbalance of TgIST-dependent inhibition of IFN-γ responses might have importance for stage conversion in neuronal cells. In the future, we plan to examine the relationship between TgIST and stage conversion in neuronal cells.

Glutamate is a major neurotransmitter; hence, brain neurons have unique glutamine metabolism (Erecińska and Silver, 1990). Glutamate and $NH_{4}^{+}$ are frequently produced by glutamine degradation in neurons. These metabolites are transported into astrocytes for glutamine synthesis by glutamine synthetase, to resupply neurons with glutamine through glutamine transporters. This process is called the glutamine-glutamate cycle (Bak et al., 2006). Previous reports have suggested a relationship between the disruption of the glutamine-glutamate cycle and *T. gondii* stage conversion. Indeed, it has been reported that the glutamine transporter function in astrocytes is inhibited by *T. gondii* infection (Lee et al., 2014), and that glutamate levels are elevated in the brain of mice with *T. gondii* chronic infection (David et al., 2016). However, the effects of *T. gondii* infection on neurons and glutamine metabolism are not well understood. In the present study, we showed that *T. gondii* infection impairs the intracellular glutamine concentration in neurons by suppressing the activation of glutamine transporters. Further investigations are needed to identify the exact virulence molecules and mechanisms involved in this phenomenon.

There are various types of neurons, including glutamatergic, GABAergic, and dopaminergic neurons; glutamatergic neurons are one of the major neurons responsible for fast excitatory transmission in the CNS (Baude et al., 2009; Langel et al., 2018). Previous studies have reported a relationship between GABAergic neurons and *T. gondii* infection (Brooks et al., 2015; Li et al., 2019), and have also reported the interaction between human neuronal-like cells and *T. gondii* bradyzoite formations (Tanaka et al., 2016; Passeri et al., 2016); however, few studies have focused on glutamatergic neurons. In the present study, we showed that *T. gondii* stage conversion is efficiently induced by IFN-γ stimulation in human iPSC-derived glutamatergic neurons, and that the mechanism involves glutamine starvation caused by glutaminase activation. Because iPSC-derived glutamatergic neurons have similar characteristics to their *in vivo* counterparts, our results suggest that glutamine starvation-induced *T. gondii* stage conversion occurs *in vivo* in humans. A previous report found that *T. gondii* mostly infects neurons and not astrocytes throughout the acute and chronic *in vivo* infection of mouse brain, and that cyst formation occurs not only in cell bodies but also in axons (Cabral et al., 2016). Our results are consistent with this previous report using mice. Together, these findings may suggest that the infection and pathology mechanisms in the brain are conserved between mice and humans even though the induction mechanisms of *T. gondii* stage conversion are different. The findings also demonstrate the importance of using model organisms and target hosts. In addition, we found that treatment with the glutaminase inhibitor CB-839 or glutaminase knockdown led to the inhibition of IFN-γ-dependent *T. gondii* stage conversion. These results support the idea that IFN-γ-dependent glutamine starvation caused by glutaminase-dependent glutamine degradation is essential for the induction of *T. gondii* stage conversion in human neuronal cells.

In summary, here we found a novel *T. gondii* stage conversion mechanism that involves IFN-γ-induced glutamine-dependent bradyzoite differentiation and cyst formation in human neuronal cells. We revealed that these effects were suppressed by treatment with a glutaminase inhibitor. Further elucidation of these effects may contribute to the development of advanced therapeutic strategies for the prevention of chronic infection of the human brain.

## Data Availability Statement

The original contributions presented in the study are included in the article/**Supplementary Material**. Further inquiries can be directed to the corresponding author.

## Author Contributions

HB and KK designed the research. HB and JO prepared the materials. HB, NW, and JO performed experiments using human cells. HB and YF set up the administrative experimental protocols for all experiments in this study. HB prepared T. gondii and performed experiments. HB and KK wrote the manuscript. KK supervised all experiments. All authors contributed to the article and approved the submitted version.

## Funding

This study was funded by grants-in-aid for Scientific Research (B:17H03913) Young Scientists (19K16628), and Young Scientists (B) (17K15677) from the Ministry of Education, Culture, Science, Sports, and Technology (MEXT) of Japan, and by a Livestock Promotional Subsidy from the Japan Racing Association, the Uehara Memorial Foundation (J200002710), and The Morinaga Foundation for Health & Nutrition (AV602061).

## Conflict of Interest

The authors declare that the research was conducted in the absence of any commercial or financial relationships that could be construed as a potential conflict of interest.

## Publisher’s Note

All claims expressed in this article are solely those of the authors and do not necessarily represent those of their affiliated organizations, or those of the publisher, the editors and the reviewers. Any product that may be evaluated in this article, or claim that may be made by its manufacturer, is not guaranteed or endorsed by the publisher.

## Supplementary Material

Supplementary Figure 1 Induction of *T. gondii* stage conversion under various experimental conditions. **(A)** HFFs, A172, U-251 MG, and IMR-32 cells infected with *T. gondii* ME49 were cultured in medium at a pH of 7.2 or 8.2. The *BAG1* mRNA level at 48 h after parasite infection was analyzed by use of quantitative RT-PCR. **(B, C)** IMR-32 cells infected with *T. gondii* strain Prugniaud were untreated or treated with IFN-γ. Cyst wall formation was assessed by IFA at 72 hours post-infection. **(B)** The percentage of CST1-positive vacuoles was determined. **(C)** Representative IFA images of *T. gondii* GAP45 (red) and CST1 (green); nuclei were stained with DAPI (blue). Scale bars correspond to 5 μm. Data are representative of three independent experiments. Indicated values are means ± SD (three biological replicates per group from three independent experiments) **(A, B)**. ***p* < 0.01; ****p* < 0.001; N.S., not significant; n.d., not detected; Student’s *t*-test.

Supplementary Figure 2 IFN-γ-induced gene expression pattern of predicted anti-*T. gondii* immune responses related to host factors in A172 and IMR-32 cells. **(A, B)** A172 and IMR-32 cells were untreated or treated with IFN-γ and incubated for 24 h. **(A)** Quantitative RT-PCR analysis of the indicated mRNA level was performed. **(B)** Heatmap of log-fold change (LFC) comparisons of differential gene expression in untreated and IFN-γ-treated A172 and IMR-32 cells. A relative decrease in LFC is indicated in blue and a relative increase is indicated in red. Values are means ± SD (three biological replicates per group from three independent experiments) **(A)**. **p* < 0.05, ****p* < 0.001; N.S., not significant; n.d., not detected; Student’s *t*-test.

Supplementary Figure 3 IFN-γ-induced metabolic profiles of *T. gondii*-infected A172 and IMR-32 cells. **(A, B)** A172 and IMR-32 cells were untreated or treated with IFN-γ and incubated for 48 h. **(A)** The levels of the indicated ions in the culture supernatant were measured by IC. **(B)** Heatmap of log-fold change (LFC) comparisons of differential ion concentrations in untreated and IFN-γ-treated A172 and IMR-32 cells. A relative decrease in LFC is indicated in blue, and an increase is indicated in red. Values are means ± SD (three biological replicates per group from three independent experiments) **(A)**. **p* < 0.05, ***p* < 0.01; N.S., not significant; Student’s *t*-test.

Supplementary Figure 4 Confirmation of differentiation of human iPSC-derived glutamatergic neurons. **(A, B)** iPSCs were differentiated for 12 days. **(A)** Morphology of neurons and complex neural networks *via* axons. Scale bars, 100 μm. **(B)** The expression of the neuronal marker TUBB3 and the glutamatergic neuron marker VGLUT1 were assessed. Representative IFA images of TUBB3 (red) or VGLUT1 (green) in iPSC-derived glutamatergic neurons; nuclei were stained with DAPI (blue). Scale bars correspond to 50 μm.

Supplementary Figure 5 The effect of glutaminase knockdown on IFN-γ-induced *T. gondii* stage conversion in human iPSC-derived glutamatergic neurons. **(A)** KGA siRNA was transfected into human iPSC-derived glutamatergic neurons. After 48 hours, the expression of KGA in human iPSC-derived glutamatergic neuron lysates was detected by Western blotting. **(B, C)** KGA siRNA was transfected into human iPSC-derived glutamatergic neurons, which were then infected with *T. gondii* ME49, and then left untreated or treated with IFN-γ. **(B)** The *BAG1* or *SAG1* mRNA level at 48 h after parasite infection was analyzed by use of quantitative RT-PCR. **(C)** The percentage of CST1-positive vacuoles in the cell bodies or axons was determined by IFA at 72 h post-infection. Values are means ± SD (three biological replicates per group from three independent experiments) **(B, C)**. **p* < 0.05, ****p* < 0.001; N.S., not significant; Student’s *t*-test.

## References

[B1] Alday P. H. Doggett J. S. (2017). Drugs in Development for Toxoplasmosis: Advances, Challenges, and Current Status. Drug Des. Devel. Ther. 11, 273–293. doi: 10.2147/dddt.S60973 PMC5279849 28182168

[B2] Bak L. K. Schousboe A. Waagepetersen H. S. (2006). The Glutamate/GABA-Glutamine Cycle: Aspects of Transport, Neurotransmitter Homeostasis and Ammonia Transfer. J. Neurochem. 98, 641–653. doi: 10.1111/j.1471-4159.2006.03913.x 16787421

[B3] Bando H. Lee Y. Sakaguchi N. Pradipta A. Ma J. S. Tanaka S.. (2018b). Inducible Nitric Oxide Synthase Is a Key Host Factor for Toxoplasma GRA15-Dependent Disruption of the Gamma Interferon-Induced Antiparasitic Human Response. MBio 9, e01738–18. doi: 10.1128/mBio.01738-18 PMC6178625 30301855

[B4] Bando H. Lee Y. Sakaguchi N. Pradipta A. Sakamoto R. Tanaka S.. (2019). Toxoplasma Effector GRA15-Dependent Suppression of IFN-γ-Induced Antiparasitic Response in Human Neurons. Front. Cell Infect. Microbiol. 9, 140. doi: 10.3389/fcimb.2019.00140 31119110 PMC6504700

[B5] Bando H. Sakaguchi N. Lee Y. Pradipta A. Ma J. S. Tanaka S.. (2018a). Toxoplasma Effector TgIST Targets Host IDO1 to Antagonize the IFN-Gamma-Induced Anti-Parasitic Response in Human Cells. Front. Immunol. 9, 2073. doi: 10.3389/fimmu.2018.02073 30283439 PMC6156249

[B6] Baude A. Strube C. Tell F. Kessler J. P. (2009). Glutamatergic Neurotransmission in the Nucleus Tractus Solitarii: Structural and Functional Characteristics. J. Chem. Neuroanat. 38, 145–153. doi: 10.1016/j.jchemneu.2009.03.004 19778680

[B7] Bierly A. L. Shufesky W. J. Sukhumavasi W. Morelli A. E. Denkers E. Y. (2008). Dendritic Cells Expressing Plasmacytoid Marker PDCA-1 are Trojan Horses During Toxoplasma Gondii Infection. J. Immunol. 181, 8485–8491. doi: 10.4049/jimmunol.181.12.8485 19050266 PMC2626190

[B8] Blume M. Rodriguez-Contreras D. Landfear S. Fleige T. Soldati-Favre D. Lucius R.. (2009). Host-Derived Glucose and its Transporter in the Obligate Intracellular Pathogen Toxoplasma Gondii are Dispensable by Glutaminolysis. Proc. Natl. Acad. Sci. U. S. A. 106, 12998–13003. doi: 10.1073/pnas.0903831106 19617561 PMC2722337

[B9] Bohne W. Heesemann J. Gross U. (1993). Induction of Bradyzoite-Specific Toxoplasma Gondii Antigens in Gamma Interferon-Treated Mouse Macrophages. Infect. Immun. 61, 1141–1145. doi: 10.1128/iai.61.3.1141-1145.1993 8432596 PMC302852

[B10] Bohne W. Heesemann J. Gross U. (1994). Reduced Replication of Toxoplasma Gondii is Necessary for Induction of Bradyzoite-Specific Antigens: A Possible Role for Nitric Oxide in Triggering Stage Conversion. Infect. Immun. 62, 1761–1767. doi: 10.1128/iai.62.5.1761-1767.1994 8168938 PMC186404

[B11] Boothroyd J. C. (2009). Toxoplasma Gondii: 25 Years and 25 Major Advances for the Field. Int. J. Parasitol. 39, 935–946. doi: 10.1016/j.ijpara.2009.02.003 19630140 PMC2895946

[B12] Braissant O. Mclin V. A. Cudalbu C. (2013). Ammonia Toxicity to the Brain. J. Inherit. Metab. Dis. 36, 595–612. doi: 10.1007/s10545-012-9546-2 23109059

[B13] Braun D. Longman R. S. Albert M. L. (2005). A Two-Step Induction of Indoleamine 2,3 Dioxygenase (IDO) Activity During Dendritic-Cell Maturation. Blood 106, 2375–2381. doi: 10.1182/blood-2005-03-0979 15947091 PMC1895261

[B14] Brooks J. M. Carrillo G. L. Su J. Lindsay D. S. Fox M. A. Blader I. J. (2015). Toxoplasma Gondii Infections Alter GABAergic Synapses and Signaling in the Central Nervous System. mBio 6, e01428–e01415. doi: 10.1128/mBio.01428-15 26507232 PMC4626855

[B15] Cabral C. M. Tuladhar S. Dietrich H. K. Nguyen E. Macdonald W. R. Trivedi T.. (2016). Neurons are the Primary Target Cell for the Brain-Tropic Intracellular Parasite Toxoplasma Gondii. PloS Pathog. 12, e1005447. doi: 10.1371/journal.ppat.1005447 26895155 PMC4760770

[B16] Cheng J. H. Xu X. Li Y. B. Zhao X. D. Aosai F. Shi S. Y.. (2020). Arctigenin Ameliorates Depression-Like Behaviors in Toxoplasma Gondii-Infected Intermediate Hosts *via* the TLR4/NF-κB and TNFR1/NF-κB Signaling Pathways. Int. Immunopharmacol. 82, 106302. doi: 10.1016/j.intimp.2020.106302 32086097

[B17] Coombes J. L. Charsar B. A. Han S. J. Halkias J. Chan S. W. Koshy A. A.. (2013). Motile Invaded Neutrophils in the Small Intestine of Toxoplasma Gondii-Infected Mice Reveal a Potential Mechanism for Parasite Spread. Proc. Natl. Acad. Sci. U.S.A. 110, E1913–E1922. doi: 10.1073/pnas.1220272110 23650399 PMC3666704

[B18] David C. N. Frias E. S. Szu J. I. Vieira P. A. Hubbard J. A. Lovelace J.. (2016). GLT-1-Dependent Disruption of CNS Glutamate Homeostasis and Neuronal Function by the Protozoan Parasite Toxoplasma Gondii. PloS Pathog. 12, e1005643. doi: 10.1371/journal.ppat.1005643 27281462 PMC4900626

[B19] Dubey J. P. (2009). History of the Discovery of the Life Cycle of Toxoplasma Gondii. Boca Raton, FL: CRC Press. Int. J. Parasitol. 39, 877–882. doi: 10.1016/j.ijpara.2009.01.005 19630138

[B20] Dubey J. P. (2010). Toxoplasmosis of Animals and Humans (CRC Press).

[B21] El Mestikawy S. Wallén-Mackenzie A. Fortin G. M. Descarries L. Trudeau L. E. (2011). From Glutamate Co-Release to Vesicular Synergy: Vesicular Glutamate Transporters. Nat. Rev. Neurosci. 12, 204–216. doi: 10.1038/nrn2969 21415847

[B22] El-On J. Peiser J. (2003). Toxoplasma and Toxoplasmosis. Harefuah 142, 48–55, 77. 12647490

[B23] Erecińska M. Silver I. A. (1990). Metabolism and Role of Glutamate in Mammalian Brain. Prog. Neurobiol. 35, 245–296. doi: 10.1016/0301-0082(90)90013-7 1980745

[B24] Ferguson D. J. Huskinson-Mark J. Araujo F. G. Remington J. S. (1994). An Ultrastructural Study of the Effect of Treatment With Atovaquone in Brains of Mice Chronically Infected With the ME49 Strain of Toxoplasma Gondii. Int. J. Exp. Pathol. 75, 111–116. 8199003 PMC2002108

[B25] Ferreira A. Caceres A. (1992). Expression of the Class III Beta-Tubulin Isotype in Developing Neurons in Culture. J. Neurosci. Res. 32, 516–529. doi: 10.1002/jnr.490320407 1527798

[B26] Ferreira-Da-Silva Mda F. Takács A. C. Barbosa H. S. Gross U. Lüder C. G. (2009). Primary Skeletal Muscle Cells Trigger Spontaneous Toxoplasma Gondii Tachyzoite-to-Bradyzoite Conversion at Higher Rates Than Fibroblasts. Int. J. Med. Microbiol. 299, 381–388. doi: 10.1016/j.ijmm.2008.10.002 19097936

[B27] Fox B. A. Gigley J. P. Bzik D. J. (2004). Toxoplasma Gondii Lacks the Enzymes Required for *De Novo* Arginine Biosynthesis and Arginine Starvation Triggers Cyst Formation. Int. J. Parasitol. 34, 323–331. doi: 10.1016/j.ijpara.2003.12.001 15003493

[B28] Frenkel J. K. Remington J. S. (1980). Hepatitis in Toxoplasmosis. N. Engl. J. Med. 302, 178–179. doi: 10.1056/NEJM198001173020316 7350455

[B29] Gomez-Marin J. E. (2000). No NO Production During Human Toxoplasma Infection. Parasitol. Today 16, 131. doi: 10.1016/S0169-4758(99)01614-2 10689335

[B30] González-González I. M. Cubelos B. Giménez C. Zafra F. (2005). Immunohistochemical Localization of the Amino Acid Transporter SNAT2 in the Rat Brain. Neuroscience 130, 61–73. doi: 10.1016/j.neuroscience.2004.09.023 15561425

[B31] Gormley P. D. Pavesio C. E. Minnasian D. Lightman S. (1998). Effects of Drug Therapy on Toxoplasma Cysts in an Animal Model of Acute and Chronic Disease. Invest. Ophthalmol. Vis. Sci. 39, 1171–1175. 9620076

[B32] Halonen S. K. Lyman W. D. Chiu F. C. (1996). Growth and Development of Toxoplasma Gondii in Human Neurons and Astrocytes. J. Neuropathol. Exp. Neurol. 55, 1150–1156. doi: 10.1097/00005072-199611000-00006 8939198

[B33] Kasper L. H. (1989). Identification of Stage-Specific Antigens of Toxoplasma Gondii. Infect. Immun. 57, 668–672. doi: 10.1128/iai.57.3.668-672.1989 2917778 PMC313159

[B34] Langel J. Ikeno T. Yan L. Nunez A. A. Smale L. (2018). Distributions of GABAergic and Glutamatergic Neurons in the Brains of a Diurnal and Nocturnal Rodent. Brain Res. 1700, 152–159. doi: 10.1016/j.brainres.2018.08.019 30153458

[B35] Lee I. P. Evans A. K. Yang C. Works M. G. Kumar V. De Miguel Z.. (2014). Toxoplasma Gondii is Dependent on Glutamine and Alters Migratory Profile of Infected Host Bone Marrow Derived Immune Cells Through SNAT2 and CXCR4 Pathways. PloS One 9, e109803. doi: 10.1371/journal.pone.0109803 25299045 PMC4192591

[B36] Li Y. Severance E. G. Viscidi R. P. Yolken R. H. Xiao J. (2019). Persistent Toxoplasma Infection of the Brain Induced Neurodegeneration Associated With Activation of Complement and Microglia. Infect. Immun. 87, e00139–19. doi: 10.1128/iai.00139-19 PMC6652752 31182619

[B37] Lüder C. G. K. Rahman T. (2017). Impact of the Host on Toxoplasma Stage Differentiation. Microb. Cell 4, 203–211. doi: 10.15698/mic2017.07.579 28706936 PMC5507683

[B38] Lyons R. E. Mcleod R. Roberts C. W. (2002). Toxoplasma Gondii Tachyzoite-Bradyzoite Interconversion. Trends Parasitol. 18, 198–201. doi: 10.1016/s1471-4922(02)02248-1 11983592

[B39] Ma J. S. Sasai M. Ohshima J. Lee Y. Bando H. Takeda K.. (2014). Selective and Strain-Specific NFAT4 Activation by the Toxoplasma Gondii Polymorphic Dense Granule Protein GRA6. J. Exp. Med. 211, 2013–2032. doi: 10.1084/jem.20131272 25225460 PMC4172224

[B40] Mayoral J. Di Cristina M. Carruthers V. B. Weiss L. M. (2020). Toxoplasma Gondii: Bradyzoite Differentiation *In Vitro* and *In Vivo* . Methods Mol. Biol. 2071, 269–282. doi: 10.1007/978-1-4939-9857-9_15 31758458 PMC7059825

[B41] Montoya J. G. Remington J. S. (2008). Management of Toxoplasma Gondii Infection During Pregnancy. Clin. Infect. Dis. 47, 554–566. doi: 10.1086/590149 18624630

[B42] Ohshima J. Lee Y. Sasai M. Saitoh T. Su Ma J. Kamiyama N.. (2014). Role of Mouse and Human Autophagy Proteins in IFN-Gamma-Induced Cell-Autonomous Responses Against Toxoplasma Gondii. J. Immunol. 192, 3328–3335. doi: 10.4049/jimmunol.1302822 24563254

[B43] Passeri E. Jones-Brando L. Bordón C. Sengupta S. Wilson A. M. Primerano A.. (2016). Infection and Characterization of Toxoplasma Gondii in Human Induced Neurons From Patients With Brain Disorders and Healthy Controls. Microbes Infect. 18, 153–158. doi: 10.1016/j.micinf.2015.09.023 26432947 PMC4758902

[B44] Pfefferkorn E. R. Rebhun S. Eckel M. (1986). Characterization of an Indoleamine 2,3-Dioxygenase Induced by Gamma-Interferon in Cultured Human Fibroblasts. J. Interferon Res. 6, 267–279. doi: 10.1089/jir.1986.6.267 2427623

[B45] Radke J. R. Donald R. G. Eibs A. Jerome M. E. Behnke M. S. Liberator P.. (2006). Changes in the Expression of Human Cell Division Autoantigen-1 Influence Toxoplasma Gondii Growth and Development. PloS Pathog. 2, e105. doi: 10.1371/journal.ppat.0020105 17069459 PMC1626100

[B46] Radke J. B. Lucas O. De Silva E. K. Ma Y. Sullivan W. J. Jr. Weiss L. M.. (2013). ApiAP2 Transcription Factor Restricts Development of the Toxoplasma Tissue Cyst. Proc. Natl. Acad. Sci. U. S. A. 110, 6871–6876. doi: 10.1073/pnas.1300059110 23572590 PMC3637731

[B47] Robert-Gangneux F. Darde M. L. (2012). Epidemiology of and Diagnostic Strategies for Toxoplasmosis. Clin. Microbiol. Rev. 25, 264–296. doi: 10.1128/cmr.05013-11 22491772 PMC3346298

[B48] Soête M. Camus D. Dubremetz J. F. (1994). Experimental Induction of Bradyzoite-Specific Antigen Expression and Cyst Formation by the RH Strain of Toxoplasma Gondii *In Vitro* . Exp. Parasitol. 78, 361–370. doi: 10.1006/expr.1994.1039 8206135

[B49] Sutterland A. L. Fond G. Kuin A. Koeter M. W. Lutter R. Van Gool T.. (2015). Beyond the Association. Toxoplasma Gondii in Schizophrenia, Bipolar Disorder, and Addiction: Systematic Review and Meta-Analysis. Acta Psychiatr. Scand. 132, 161–179. doi: 10.1111/acps.12423 25877655

[B50] Swierzy I. J. Lüder C. G. (2015). Withdrawal of Skeletal Muscle Cells From Cell Cycle Progression Triggers Differentiation of Toxoplasma Gondii Towards the Bradyzoite Stage. Cell Microbiol. 17, 2–17. doi: 10.1111/cmi.12342 25131712

[B51] Tanaka N. Ashour D. Dratz E. Halonen S. (2016). Use of Human Induced Pluripotent Stem Cell-Derived Neurons as a Model for Cerebral Toxoplasmosis. Microbes Infect. 18, 496–504. doi: 10.1016/j.micinf.2016.03.012 27083472

[B52] Tobin C. Pollard A. Knoll L. (2010). Toxoplasma Gondii Cyst Wall Formation in Activated Bone Marrow-Derived Macrophages and Bradyzoite Conditions. J. Vis. Exp. 42, 2091. doi: 10.3791/2091 PMC3156017 20736916

[B53] Tomavo S. Fortier B. Soete M. Ansel C. Camus D. Dubremetz J. F. (1991). Characterization of Bradyzoite-Specific Antigens of Toxoplasma Gondii. Infect. Immun. 59, 3750–3753. doi: 10.1128/iai.59.10.3750-3753.1991 1894373 PMC258946

[B54] Tomita T. Bzik D. J. Ma Y. F. Fox B. A. Markillie L. M. Taylor R. C.. (2013). The Toxoplasma Gondii Cyst Wall Protein CST1 is Critical for Cyst Wall Integrity and Promotes Bradyzoite Persistence. PloS Pathog. 9, e1003823. doi: 10.1371/journal.ppat.1003823 24385904 PMC3873430

[B55] Umar S. van der Laarse A. (2010). Nitric Oxide and Nitric Oxide Synthase Isoforms in the Normal, Hypertrophic, and Failing Heart. Mol. Cell Biochem. 333, 191–201. doi: 10.1007/s11010-009-0219-x 19618122

[B56] Unno A. Suzuki K. Xuan X. Nishikawa Y. Kitoh K. Takashima Y. (2008). Dissemination of Extracellular and Intracellular Toxoplasma Gondii Tachyzoites in the Blood Flow. Parasitol. Int. 57, 515–518. doi: 10.1016/j.parint.2008.06.004 18652914

[B57] Varoqui H. Zhu H. Yao D. Ming H. Erickson J. D. (2000). Cloning and Functional Identification of a Neuronal Glutamine Transporter. J. Biol. Chem. 275, 4049–4054. doi: 10.1074/jbc.275.6.4049 10660562

[B58] Waldman B. S. Schwarz D. Wadsworth M. H. 2nd Saeij J. P. Shalek A. K. Lourido S. (2020). Identification of a Master Regulator of Differentiation in Toxoplasma. Cell 180, 359–372.e316. doi: 10.1016/j.cell.2019.12.013 31955846 PMC6978799

[B59] Wang F. Chen S. Jiang Y. Zhao Y. Sun L. Zheng B.. (2018). Effects of Ammonia on Apoptosis and Oxidative Stress in Bovine Mammary Epithelial Cells. Mutagenesis 33, 291–299. doi: 10.1093/mutage/gey023 30184101

[B60] Watts E. Zhao Y. Dhara A. Eller B. Patwardhan A. Sinai A. P. (2015). Novel Approaches Reveal That Toxoplasma Gondii Bradyzoites Within Tissue Cysts Are Dynamic and Replicating Entities *In Vivo* . mBio 6, e01155–e01115. doi: 10.1128/mBio.01155-15 26350965 PMC4600105

[B61] Weiss L. M. Laplace D. Takvorian P. M. Tanowitz H. B. Cali A. Wittner M. (1995). A Cell Culture System for Study of the Development of Toxoplasma Gondii Bradyzoites. J. Eukaryot. Microbiol. 42, 150–157. doi: 10.1111/j.1550-7408.1995.tb01556.x 7757057

[B62] Xu X. Meng Y. Li L. Xu P. Wang J. Li Z.. (2019). Overview of the Development of Glutaminase Inhibitors: Achievements and Future Directions. J. Med. Chem. 62, 1096–1115. doi: 10.1021/acs.jmedchem.8b00961 30148361

[B63] Yamamoto M. Okuyama M. Ma J. S. Kimura T. Kamiyama N. Saiga H.. (2012). A Cluster of Interferon-Gamma-Inducible P65 GTPases Plays a Critical Role in Host Defense Against Toxoplasma Gondii. Immunity 37, 302–313. doi: 10.1016/j.immuni.2012.06.009 22795875

[B64] Yap G. S. Scharton-Kersten T. Charest H. Sher,. A. (1998). Decreased Resistance of TNF Receptor P55- and P75-Deficient Mice to Chronic Toxoplasmosis Despite Normal Activation of Inducible Nitric Oxide Synthase *In Vivo* . J. Immunol. 160, 1340–1345. 9570552

[B65] Zeng H. Sanes J. R. (2017). Neuronal Cell-Type Classification: Challenges, Opportunities and the Path Forward. Nat. Rev. Neurosci. 18, 530–546. doi: 10.1038/nrn.2017.85 28775344

[B66] Zhang Y. W. Smith J. E. (1995). Toxoplasma Gondii: Identification and Characterization of a Cyst Molecule. Exp. Parasitol. 80, 228–233. doi: 10.1006/expr.1995.1028 7534723

