# Supplementary figures and images for: Depletion of Intracellular Glutamine Pools Triggers Toxoplasma gondii Stage Conversion in Human Glutamatergic Neurons
Source: Front Cell Infect Microbiol. 2022 Jan 13;11:788303. doi: 10.3389/fcimb.2021.788303 (PMC8793678; doi:10.3389/fcimb.2021.788303)

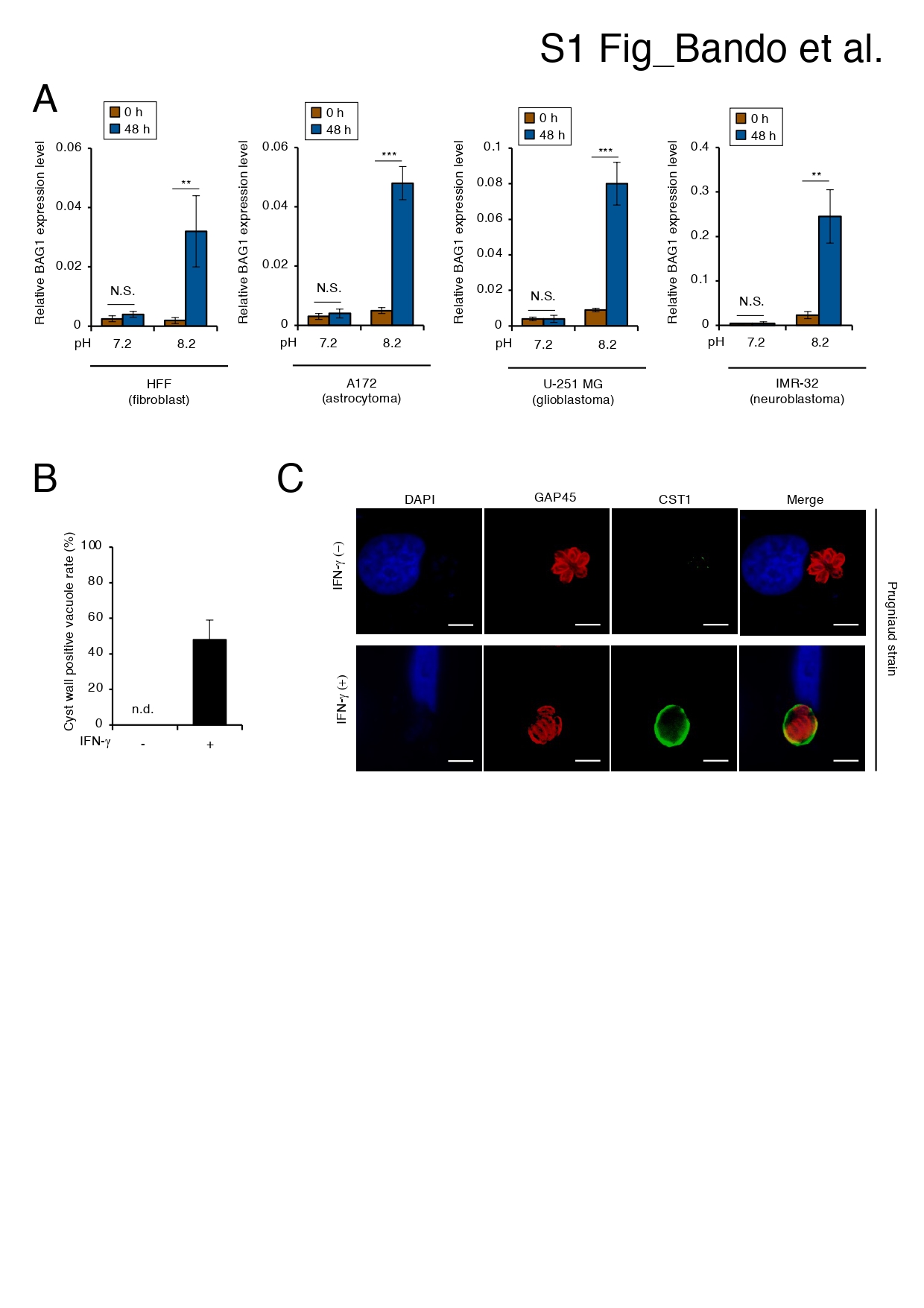

Supplement: Supplementary Figure 1 — Induction of T. gondii stage conversion under various experimental conditions. (A) HFFs, A172, U-251 MG, and IMR-32 cells infected with T. gondii ME49 were cultured in medium at a pH of 7.2 or 8.2. The BAG1 mRNA level at 48 h after parasite infection was analyzed by use of quantitative RT-PCR. (B, C) IMR-32 cells infected with T. gondii strain Prugniaud were untreated or treated with IFN-γ. Cyst wall formation was assessed by IFA at 72 hours post-infection. (B) The percentage of CST1-positive vacuoles was determined. (C) Representative IFA images of T. gondii GAP45 (red) and CST1 (green); nuclei were stained with DAPI (blue). Scale bars correspond to 5 μm. Data are representative of three independent experiments. Indicated values are means ± SD (three biological replicates per group from three independent experiments) (A, B). **p < 0.01; ***p < 0.001; N.S., not significant; n.d., not detected; Student’s t-test. [file Image_1.jpeg]

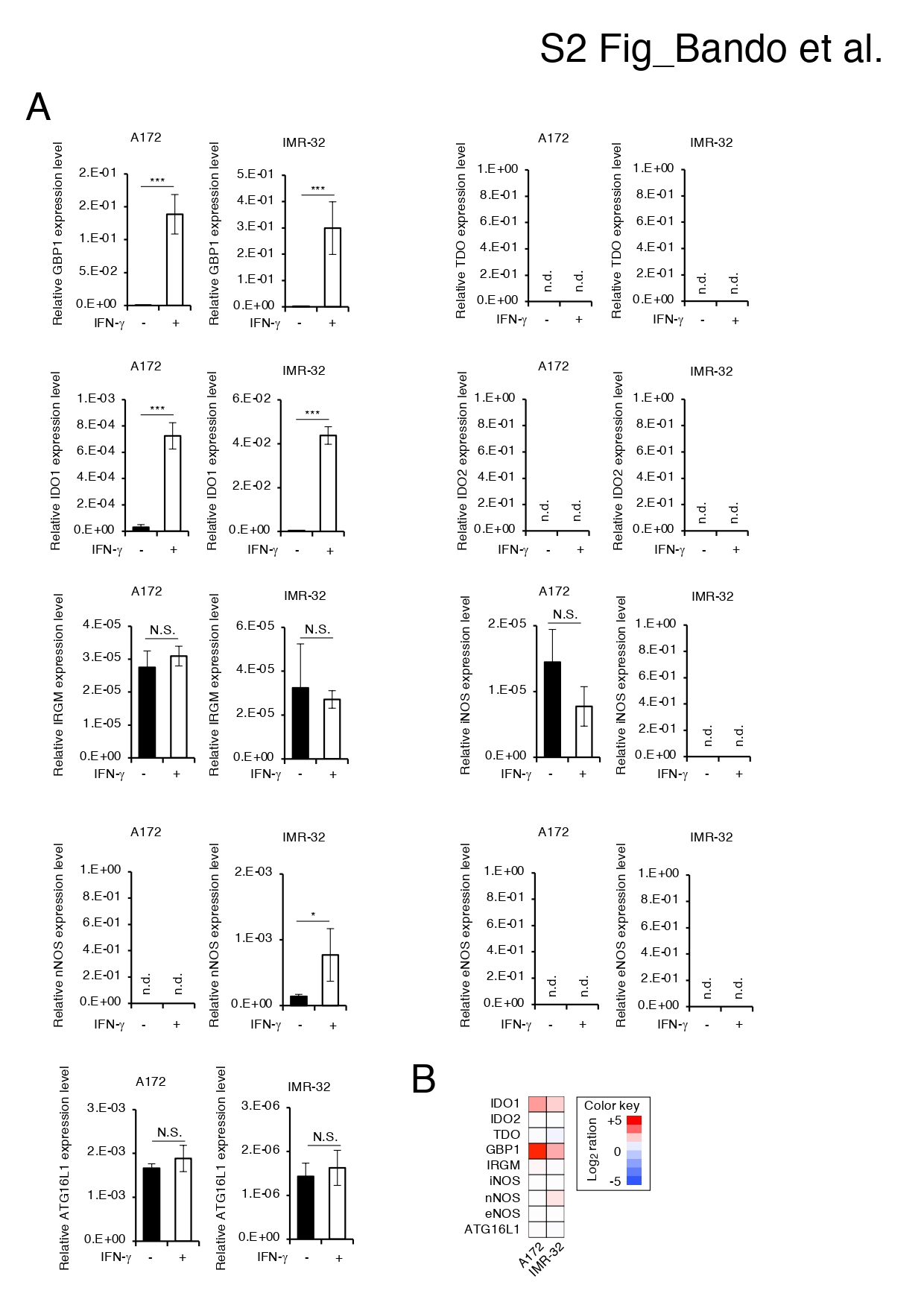

Supplement: Supplementary Figure 2 — IFN-γ-induced gene expression pattern of predicted anti-T. gondii immune responses related to host factors in A172 and IMR-32 cells. (A, B) A172 and IMR-32 cells were untreated or treated with IFN-γ and incubated for 24 h. (A) Quantitative RT-PCR analysis of the indicated mRNA level was performed. (B) Heatmap of log-fold change (LFC) comparisons of differential gene expression in untreated and IFN-γ-treated A172 and IMR-32 cells. A relative decrease in LFC is indicated in blue and a relative increase is indicated in red. Values are means ± SD (three biological replicates per group from three independent experiments) (A). *p < 0.05, ***p < 0.001; N.S., not significant; n.d., not detected; Student’s t-test. [file Image_2.jpeg]

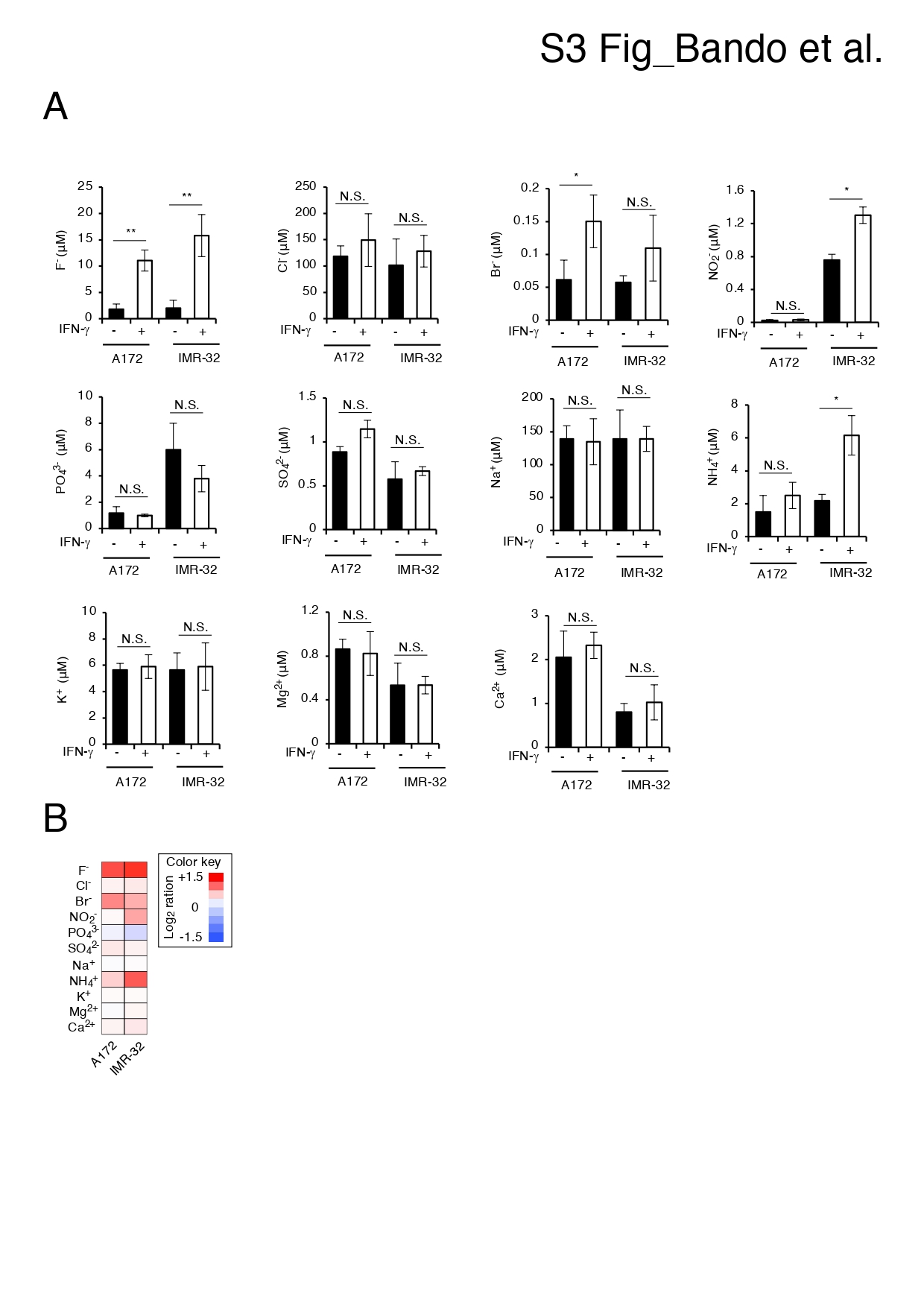

Supplement: Supplementary Figure 3 — IFN-γ-induced metabolic profiles of T. gondii-infected A172 and IMR-32 cells. (A, B) A172 and IMR-32 cells were untreated or treated with IFN-γ and incubated for 48 h. (A) The levels of the indicated ions in the culture supernatant were measured by IC. (B) Heatmap of log-fold change (LFC) comparisons of differential ion concentrations in untreated and IFN-γ-treated A172 and IMR-32 cells. A relative decrease in LFC is indicated in blue, and an increase is indicated in red. Values are means ± SD (three biological replicates per group from three independent experiments) (A). *p < 0.05, **p < 0.01; N.S., not significant; Student’s t-test. [file Image_3.jpeg]

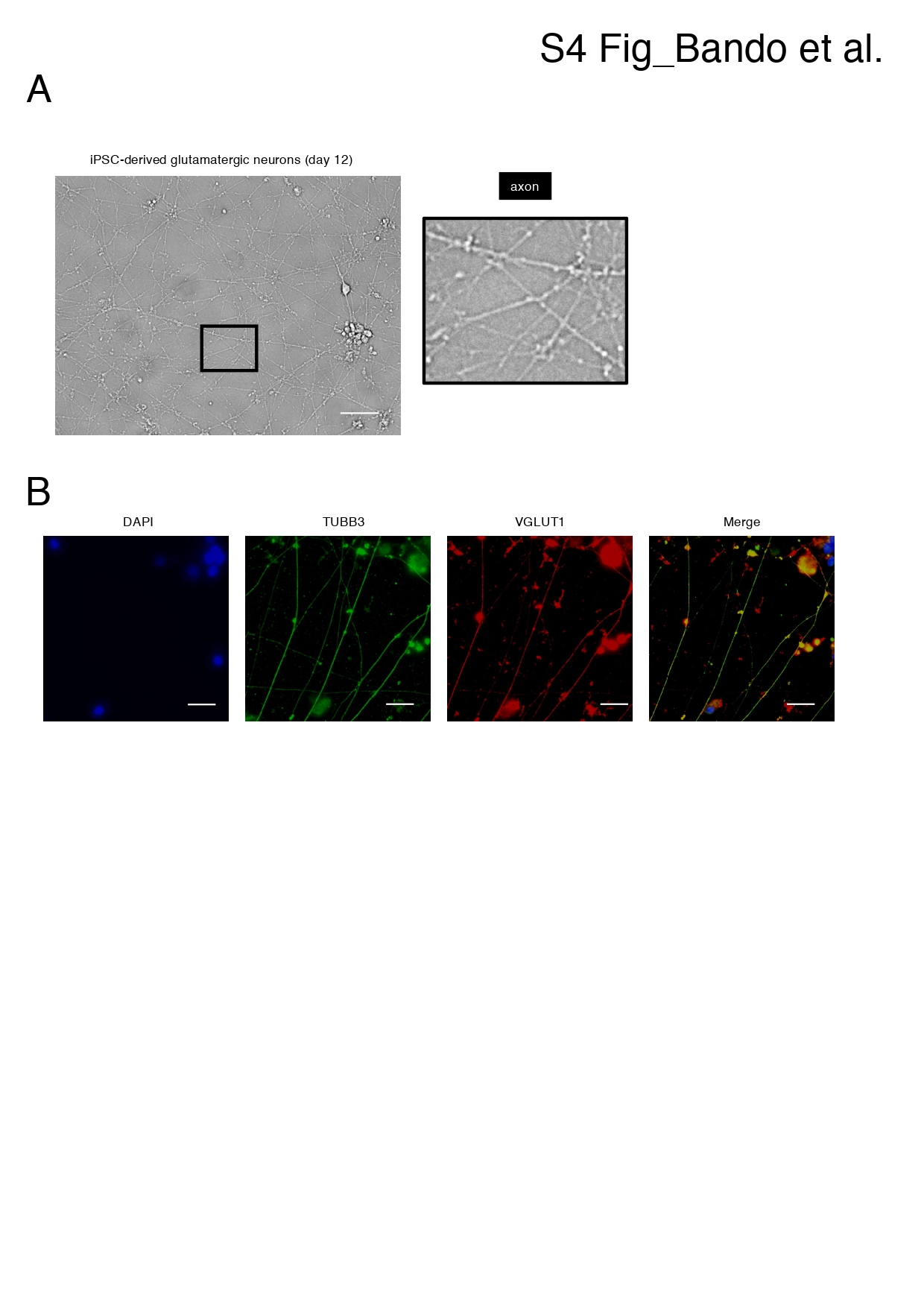

Supplement: Supplementary Figure 4 — Confirmation of differentiation of human iPSC-derived glutamatergic neurons. (A, B) iPSCs were differentiated for 12 days. (A) Morphology of neurons and complex neural networks via axons. Scale bars, 100 μm. (B) The expression of the neuronal marker TUBB3 and the glutamatergic neuron marker VGLUT1 were assessed. Representative IFA images of TUBB3 (red) or VGLUT1 (green) in iPSC-derived glutamatergic neurons; nuclei were stained with DAPI (blue). Scale bars correspond to 50 μm. [file Image_4.jpeg]

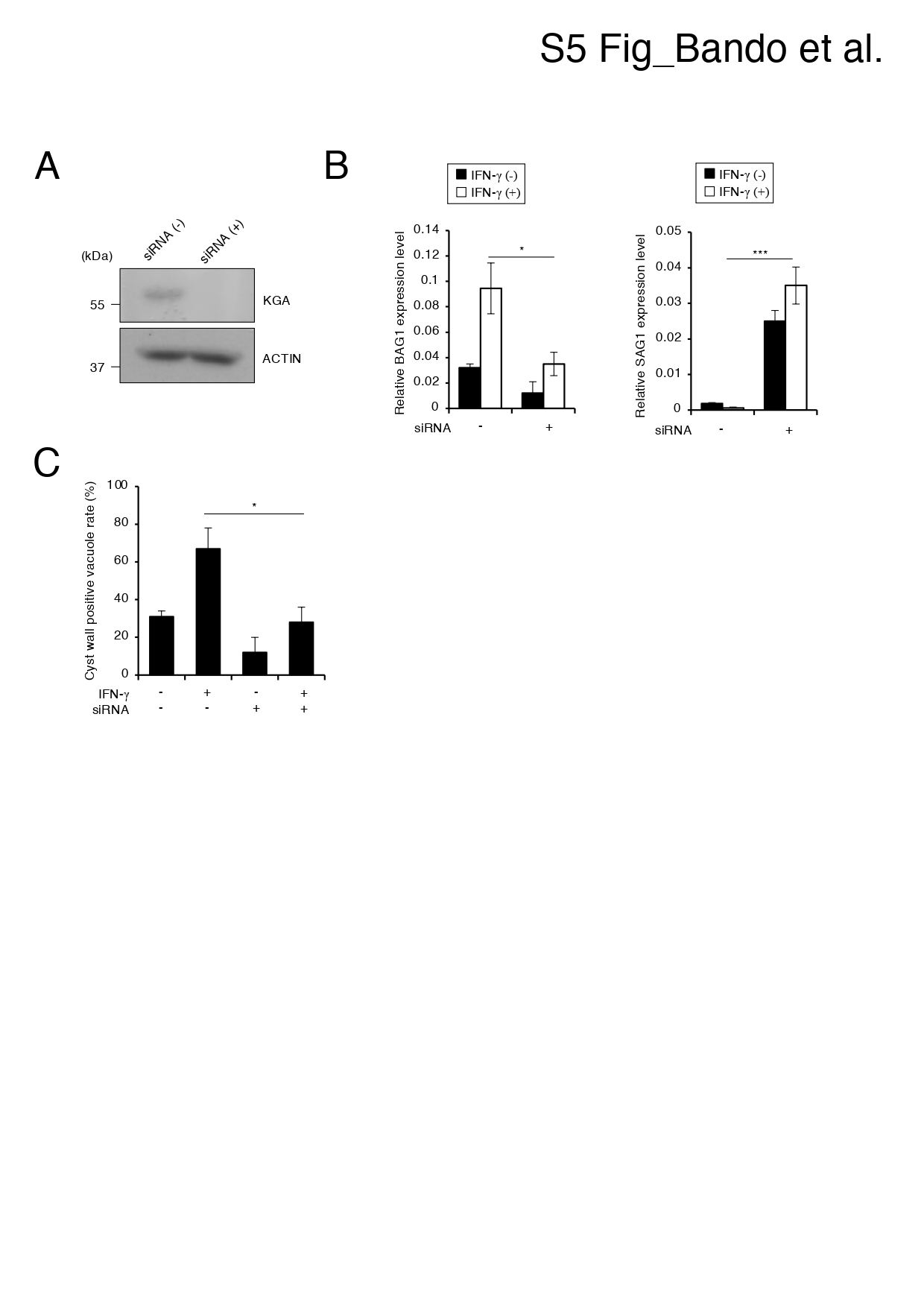

Supplement: Supplementary Figure 5 — The effect of glutaminase knockdown on IFN-γ-induced T. gondii stage conversion in human iPSC-derived glutamatergic neurons. (A) KGA siRNA was transfected into human iPSC-derived glutamatergic neurons. After 48 hours, the expression of KGA in human iPSC-derived glutamatergic neuron lysates was detected by Western blotting. (B, C) KGA siRNA was transfected into human iPSC-derived glutamatergic neurons, which were then infected with T. gondii ME49, and then left untreated or treated with IFN-γ. (B) The BAG1 or SAG1 mRNA level at 48 h after parasite infection was analyzed by use of quantitative RT-PCR. (C) The percentage of CST1-positive vacuoles in the cell bodies or axons was determined by IFA at 72 h post-infection. Values are means ± SD (three biological replicates per group from three independent experiments) (B, C). *p < 0.05, ***p < 0.001; N.S., not significant; Student’s t-test. [file Image_5.jpeg]

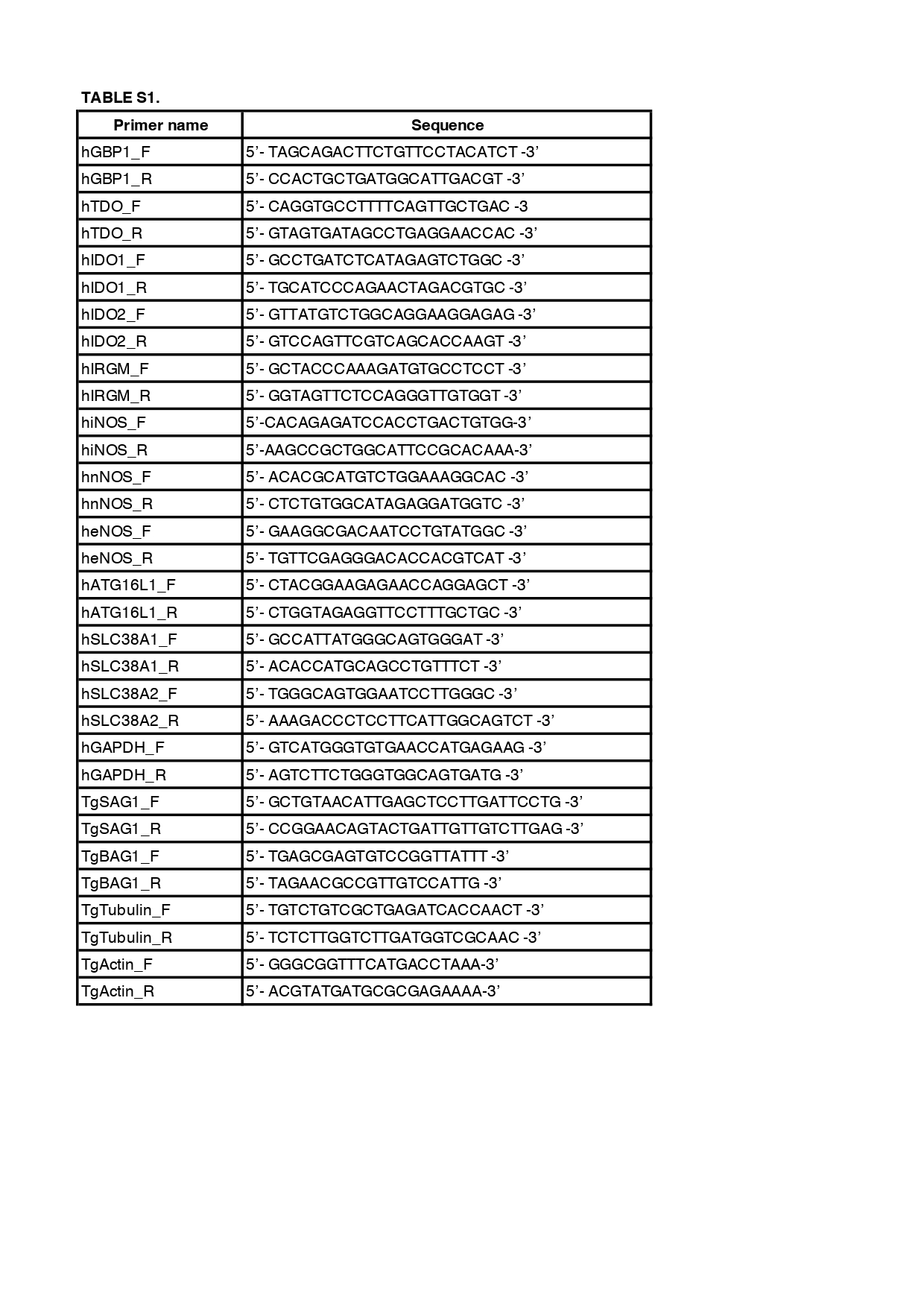

Supplement: Supplementary file 6 [file Image_6.jpeg]
